# Comparative efficacy and safety of adjunctive drugs to levodopa for fluctuating Parkinson’s disease - network meta-analysis

**DOI:** 10.1038/s41531-023-00589-8

**Published:** 2023-10-19

**Authors:** Wataru Sako, Yuki Kogo, Michinori Koebis, Yoshiaki Kita, Hajime Yamakage, Takayuki Ishida, Nobutaka Hattori

**Affiliations:** 1 Department of Neurology, Juntendo University Graduate School of Medicine, Tokyo, Japan; 2 Medical Headquarters, Eisai Co., Ltd., Tokyo, Japan; 3 Publication Business, Medical Professional Relations Inc., Osaka, Japan; 4 Department of Medical Statistics, Satista Co., Ltd., Kyoto, Japan

**Keywords:** Parkinson's disease, Drug therapy

## Abstract

It remains unclear which adjunctive drug for Parkinson’s disease (PD) in combination with levodopa is more effective, tolerable, and safe. We aimed to compare the efficacy, tolerability, and safety among anti-PD drugs from several classes in patients with fluctuating PD who received levodopa through network meta-analysis (NMA). Twelve anti-PD drugs belonging to 4 different drug classes (dopamine agonists, monoamine oxidase type B inhibitors, catechol-O-methyl transferase inhibitors, and an adenosine A2A receptor antagonist) were selected. We systematically searched PubMed, Embase, and the Cochrane Library for eligible randomized controlled trials (RCTs) comparing placebo with anti-PD drug or among anti-PD drugs in patients with PD who experienced motor fluctuations or wearing-off and received levodopa. We included 54 RCTs in the analysis. The NMA was performed under a frequentist framework using a random-effects model. The efficacy outcome was change in daily off-time, and the tolerability outcome was discontinuation due to all causes. Safety outcomes included discontinuation due to adverse events (AEs) and the incidence of AEs, dyskinesia, hallucination, and orthostatic hypotension. According to the surface under the cumulative ranking curve (SUCRA) in the NMA, ropinirole transdermal patch (SUCRA, 0.861) ranked the highest in efficacy, followed by pramipexole (0.762), ropinirole extended release (ER) (0.750), and safinamide (0.691). In terms of tolerability, ropinirole (0.954) ranked the highest, followed by pramipexole (0.857), safinamide (0.717), and ropinirole ER (0.708). Each anti-PD drug had different SUCRA ranking profiles for the safety outcomes. These findings suggest that ropinirole, pramipexole, and safinamide are well-balanced anti-PD drugs that satisfy both efficacy and tolerability outcomes.

## Introduction

Parkinson’s disease (PD) is a neurodegenerative disease characterized by motor symptoms and pathological feature of loss of dopaminergic neurons in the substantia nigra^1,2^. The gold standard for PD treatment is dopamine replacement therapy with levodopa, but its long-term use often results in complications such as motor fluctuations, wearing-off, and dyskinesia^3^. Patients with PD who experience these complications often receive other anti-PD drugs in combination with levodopa, including dopamine agonists (DAs), monoamine oxidase type B inhibitors (MAOBIs), catechol-O-methyl transferase inhibitors (COMTIs), and adenosine A2A receptor antagonist (A2ARA)^2,3^.

All the approved anti-PD drugs have been reported to be effective for reducing daily off-time in patients with PD and motor fluctuations (fluctuating PD) who received levodopa, while each of these drugs has a specific risk profile for adverse events (AEs), such as dyskinesia, hallucination, and orthostatic hypotension (OH)^2,3^. Therefore, physicians need to select the most appropriate anti-PD drug from many options to manage each patient.

Many randomized controlled trials (RCTs) have evaluated the efficacy and safety of anti-PD drugs. However, only a few trials have directly compared anti-PD drugs. A network meta-analysis (NMA) allows for the comparison of outcomes among two or more active treatments in a network of studies, even if there is no direct comparison between each treatment^4^. Previously, some reports applying NMAs have demonstrated comparative results among anti-PD drugs^5–13^. Many of them included intra-drug class comparisons, and NMAs that compared anti-PD drugs from different drug classes are limited. Zhuo et al. reported an NMA that compared anti-PD drugs from different drug classes^5^, but it didn’t include istradefylline, safinamide, or COMTIs, which have become recently available, nor did it distinguish DA dosage forms or progression stages of the disease. In addition, previous NMAs including this used Unified Parkinson’s Disease Rating Scale (UPDRS) scores^5,6^ to assess efficacy. However, a reduction in daily off-time is generally used to evaluate the efficacy of levodopa adjunctive drugs in RCTs and may be sometimes more important for patients with advanced PD. Therefore, an NMA that focuses on motor fluctuation in advanced PD is warranted.

In this study, we performed a systematic review and NMA to compare the efficacy, tolerability, and safety among anti-PD drugs adjunct to levodopa in patients with fluctuating PD. We focused on the following 12 anti-PD drugs approved in Japan: pramipexole, pramipexole extended release (ER), ropinirole, ropinirole ER, ropinirole transdermal patch, rotigotine transdermal patch (all DAs); rasagiline, safinamide, and selegiline (all MAOBIs); entacapone and opicapone (both COMTIs); and istradefylline (an A2ARA). We used the change in daily off-time to evaluate efficacy, discontinuation due to all causes to evaluate tolerability (against efficacy and/or safety problems), and discontinuation due to AEs and the incidence of AEs, dyskinesia, hallucination, and OH to evaluate safety.

## Results

### Study characteristics

**Fig. 1:**
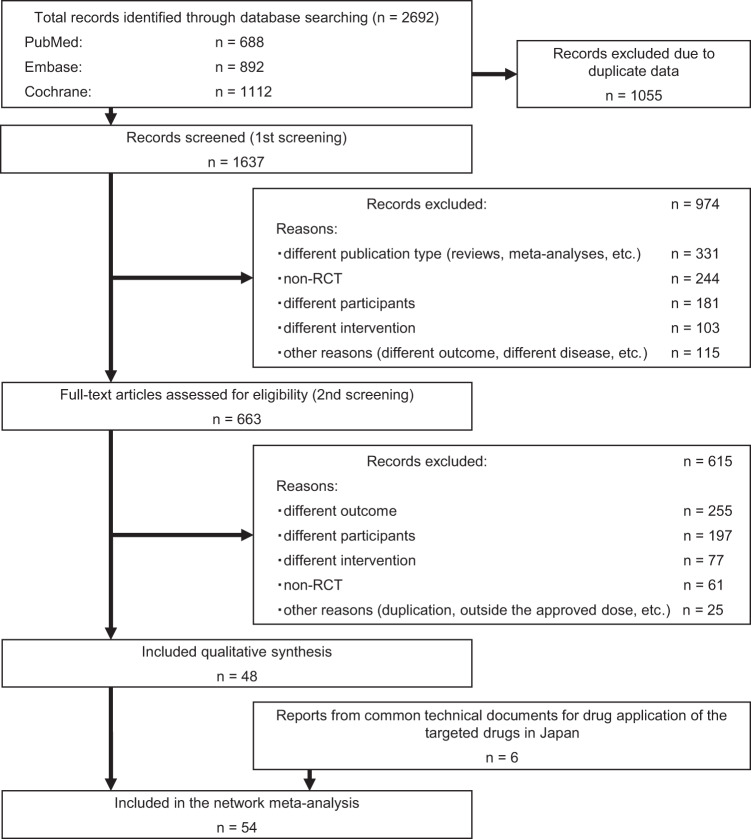
Flow diagram of the literature search and selection process. RCT randomized controlled clinical trial.

**Fig. 2:**
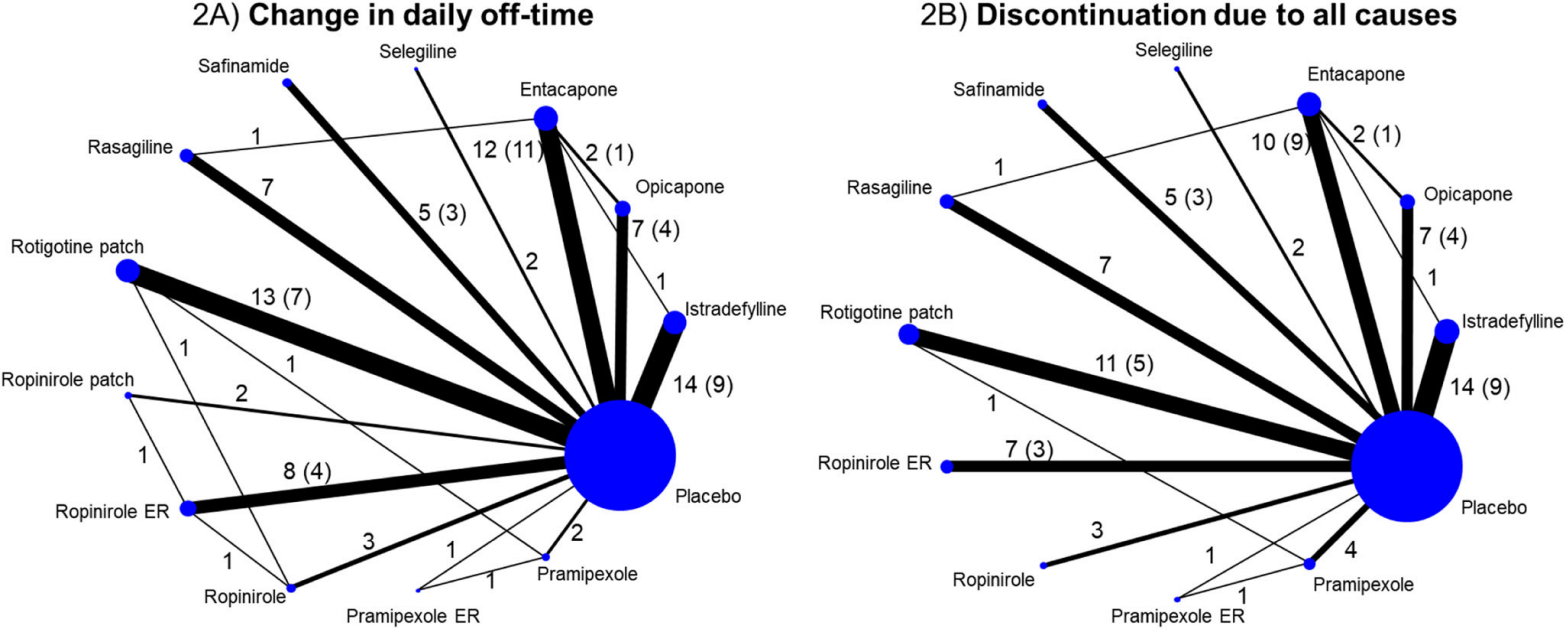
Network maps of the included trials for change in daily off-time and discontinuation due to all causes. (**A**) Change in daily off-time, (**B**) Discontinuation due to all causes. The circular nodes indicate each treatment. The size of the nodes corresponds to the number of patients assigned to each treatment. Treatments with direct comparisons are linked with a line, and the thickness of the line corresponds to the number of comparisons. The figure next to the line shows the number of comparisons and the figure in parentheses shows the number of trials if there are differences between the number of comparisons and trials. ER extended release, ropinirole patch ropinirole transdermal patch, rotigotine patch rotigotine transdermal patch.

A flowchart of the literature screening process is shown in Fig. 1. The initial search yielded 2692 records, 1637 of which remained after the duplicates were removed. After the titles and abstracts were reviewed, followed by full-text reviews, an additional 1589 articles did not meet the inclusion criteria and were thus excluded. Thus, 48 studies^14–61^ were included in the NMA. Characteristics of the included studies are shown in Supplementary Table 1, and the risk of bias assessment is presented in Supplementary Fig. 1. In addition to these 48 studies, 6 eligible reports from Japanese common technical documents (CTD) were also included. Thus, the NMA included a total of 54 studies involving 12 different drugs for PD. However, only 11 drugs were included in the tolerability and safety outcome NMA given the lack of information available on the ropinirole transdermal patch. A network map of the included studies for each outcome is shown in Fig. 2 or Supplementary Fig. 2.

### NMA results

**Fig. 3:**
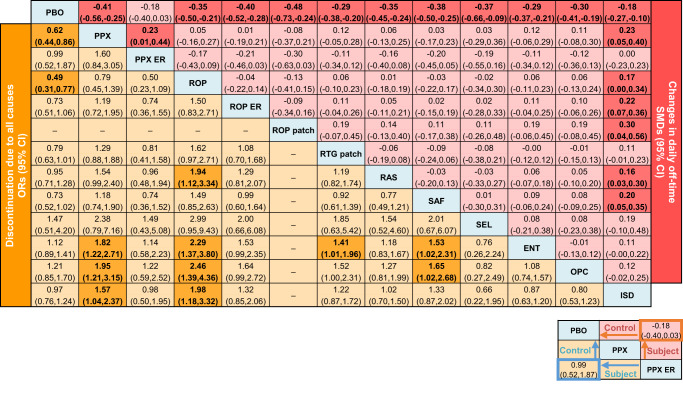
League tables of the NMA results for change in daily off-time and discontinuation due to all causes. The numbers on the right upper triangle are SMDs (95% CIs) for the change in daily off-time in the column-defining treatment compared with the row-defining treatment, and smaller SMD values than 0 indicate greater effect. Those on the left bottom triangle represent the ORs (95% CIs) of discontinuation due to all causes in the row-defining treatment compared with the column-defining treatment, and smaller OR values than 1 indicate lower risk. All numbers are shown after rounding off to two decimal places. 0.00 and −0.00 mean positive and negative values, respectively. The bold font indicates significant results. In the global inconsistency test, Chi-square = 3.04 and *P* = 0.932 for change in daily off-time and Chi-square = 9.04 and *P* = 0.107 for discontinuation due to all causes. No inconsistency was detected in either NMA. NMA network meta-analysis, PBO placebo, PPX pramipexole, PPX ER pramipexole extended release, ROP ropinirole, ROP ER ropinirole extended release, ROP patch ropinirole transdermal patch, RTG patch rotigotine transdermal patch, RAS rasagiline, SAF safinamide, SEL selegiline, ENT entacapone, OPC opicapone, ISD istradefylline, SMD standardized mean difference, CI confidence interval, OR odds ratio.

**Fig. 4:**
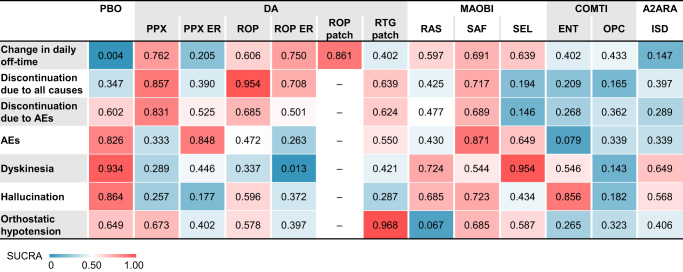
Heat map of each outcome based on the surface under the cumulative ranking curve (SUCRA). The SUCRA values are shown in each box. Red indicates a higher SUCRA value with a greater probability of being the best treatment, and blue indicates a lower SUCRA value with a lower probability of being the best treatment. DA dopamine agonist, MAOBI monoamine oxidase type B inhibitor, COMTI catechol-O-methyl transferase inhibitor, A2ARA adenosine A2A receptor antagonist, PBO placebo, PPX pramipexole, PPX ER pramipexole extended release, ROP ropinirole, ROP ER ropinirole extended-release, ROP patch ropinirole transdermal patch, RTG patch rotigotine transdermal patch, RAS rasagiline, SAF safinamide, SEL selegiline, ENT entacapone, OPC opicapone, ISD istradefylline, AE adverse event.

Figure 3 shows the NMA results for the change in daily off-time and the risk of discontinuation due to all causes. NMA results for the other outcomes are shown in Supplementary Fig. 3. Supplementary Fig. 4 shows forest plots for each outcome to illustrate the effect of each study, the pooled effect of all studies in the direct comparison, and the pooled effect of all studies in the NMA along with the 95% confidence intervals (CIs). The heterogeneity of the included studies and the inconsistency of the analysis for each outcome are summarized in Supplementary Table 2 and Supplementary Table 3, respectively. Supplementary Fig. 5 shows the rank probability curves, and Fig. 4 summarizes the surface under the cumulative ranking curve (SUCRA) values of comparable treatments for all outcomes.

In terms of the change in daily off-time, all drugs except for pramipexole ER (standardized mean difference (SMD) [pramipexole ER–placebo], 95% CI: −0.18, −0.40 to 0.03) showed significant improvement compared with placebo (Fig. 3, Supplementary Fig. 3A, 4A). According to the SUCRA, ropinirole transdermal patch (SUCRA, 0.861) ranked the highest in change in daily off-time, followed by pramipexole (0.762), ropinirole ER (0.750), and safinamide (0.691) (Fig. 4, Supplementary Fig. 5A).

In terms of discontinuation due to all causes, pramipexole (odds ratio (OR), 95% CI: 0.62, 0.44 to 0.86) and ropinirole (0.49, 0.31 to 0.77) were associated with a significantly lower risk compared with placebo (Fig. 3, Supplementary Fig. 3B, 4B). According to the SUCRA, ropinirole (SUCRA, 0.954) ranked the highest, followed by pramipexole (0.857), safinamide (0.717), and ropinirole ER (0.708) (Fig. 4, Supplementary Fig. 5B).

In terms of discontinuation due to AEs, none of the 11 drugs were associated with a lower risk compared with placebo (Supplementary Fig. 3C, 4C). According to the SUCRA, pramipexole (SUCRA, 0.831) ranked the highest, followed by safinamide (0.689), ropinirole (0.685), and rotigotine transdermal patch (0.624) (Fig. 4, Supplementary Fig. 5C).

In terms of the incidence of AEs, none of the 11 drugs were associated with a lower risk compared with placebo (Supplementary Fig. 3D, 4D). According to the SUCRA, safinamide (SUCRA, 0.871) ranked the highest, followed by pramipexole ER (0.848), selegiline (0.649), and rotigotine transdermal patch (0.550) (Fig. 4, Supplementary Fig. 5D).

In terms of the incidence of dyskinesia, none of the 11 drugs were associated with a lower risk compared with placebo (Supplementary Fig. 3E, 4E). According to the SUCRA, selegiline (SUCRA, 0.954) ranked the highest, followed by rasagiline (0.724), istradefylline (0.649), and entacapone (0.546) (Fig. 4, Supplementary Fig. 5E).

In terms of the incidence of hallucination, none of the 11 drugs were associated with a lower risk compared with placebo (Supplementary Fig. 3F, 4F). According to the SUCRA, entacapone (SUCRA, 0.856) ranked the highest, followed by safinamide (0.723), rasagiline (0.685), and ropinirole (0.596) (Fig. 4, Supplementary Fig. 5F).

In terms of the incidence of OH, rotigotine transdermal patch (OR, 95% CI: 0.40, 0.24 to 0.68) was associated with a lower risk compared with placebo (Supplementary Fig. 3G, 4G). According to the SUCRA, rotigotine transdermal patch (SUCRA, 0.968) ranked the highest, followed by safinamide (0.685), pramipexole (0.673), and selegiline (0.587) (Fig. 4, Supplementary Fig. 5G).

### Inconsistency assessment

No global inconsistency of treatment effect was found for any of the outcomes (all *P* > 0.05) (Fig. 3, Supplementary Fig. 3). Additionally, no significant inconsistency was observed between the direct and indirect studies for any of the outcomes, except for the incidence of discontinuation due to all causes (Supplementary Table 3). In the network for the incidence of discontinuation due to all causes, an inconsistency between the direct and indirect comparison of entacapone and placebo was detected (Supplementary Table 3B, *P* = 0.018).

## Discussion

This is the NMA that evaluated and compared anti-PD drugs from several drug classes in patients with fluctuating PD. We evaluated change in daily off-time, a common clinical measure in the treatment of fluctuating PD, as an efficacy outcome. We also evaluated 1 tolerability outcome (discontinuation due to all causes) and 5 safety outcomes (discontinuation due to AEs and the incidence of AEs, dyskinesia, hallucination, and OH), all of which are important for physicians to consider when selecting appropriate anti-PD drugs for individual patients.

The SMDs of our NMA demonstrated that adjunctive anti-PD drugs were generally more effective than placebo in reducing the daily off time. Except for pramipexole ER and istradefylline, no medication displayed a statistically significant difference in efficacy when compared with other treatments. However, the SUCRA values for efficacy varied among the drugs, with the highest value being 0.861 for ropinirole transdermal patch. Pramipexole (0.762), ropinirole ER (0.750), safinamide (0.691), and selegiline (0.639) came next in that order, while opicapone (0.433), entacapone (0.402), and istradefylline (0.147) ranked lower. These results suggest that DAs and MAOBIs are more effective than COMTIs and an A2ARA.

Although most DAs showed higher SUCRA values for efficacy outcome, pramipexole ER and rotigotine transdermal patch ranked lower among the 12 anti-PD drugs. The low ranking of pramipexole ER might be resulted from the fact that our analysis included only one relevant RCT, which showed that pramipexole ER had lower efficacy than pramipexole^52^. For rotigotine transdermal patch, our result was inconsistent with previous NMA studies in terms of relative efficacy ranking to ropinirole^10,13^. The discrepancy may be caused by SP511 study, which was included only in the present study (Supplementary Fig. 4A). This was an unpublished phase 2b, 12 weeks, multi-center, double-blind, randomized, placebo-controlled dose-ranging trial^62,63^. A total of 324 patients were allocated to four arms: placebo or rotigotine 4, 8, 12 mg/24 h^62,63^. This study showed great improvement in the active arms and in the placebo arm, so there were no significant differences between the drugs and placebo. Some other included studies were different between the present and previous studies^10,13^; nevertheless, in all three NMA studies, pramipexole and ropinirole ER ranked higher, suggesting the robustness of the findings of the present analysis.

Among the MAOBIs, safinamide ranked highest in terms of efficacy, followed by selegiline and rasagiline. There have already been two reports of NMAs focusing on MAOBIs, and the relative ranking of these drugs varied between studies, including the present study^7,12^. Differences in efficacy outcomes could be one reason for this discrepancy. The NMA reported by Binde et al.^7^ used responder rate defined according to the Clinical Global Impression scale or UPDRS score as the efficacy outcome, whereas the present study and another NMA^12^ used off-time. Because most recent clinical trials in patients with fluctuating PD used patient diary as the primary endpoint, off-time must be more useful to evaluate the clinical efficacy of anti-PD drugs for fluctuating PD. The effect size was the mean difference in a previous report^12^, and we used SMDs as the effect size, which is widely used and recommended in the Cochrane Handbook^64^. In the present study and in a previous study that used off-time as an efficacy outcome^12^, selegiline and safinamide had higher SUCRA values than the other MAOBI. These analyses included two studies of selegiline, both of which used doses that were lower than the maximal approved dose (up to 2.5 mg/day vs. 10 mg/day). Therefore, the efficacy of selegiline might have been underestimated. Since there was no statistically significant difference in the reduction of off-time between the MAOBIs in the present and previous studies^12^, no conclusion about the differences in the efficacy of MAOBIs can be made. These three drugs ranked in a similar order among the 12 anti-PD drugs examined in this study, demonstrating the relative efficacy of this drug class.

Among the COMTIs, opicapone ranked higher than entacapone for efficacy. Consistent with our results, a previously reported NMA that compared the efficacy of COMTIs in patients with fluctuating PD who received levodopa found that opicapone (SUCRA, 0.5942) ranked higher than entacapone (0.4038) in terms of extending the on-time^11^.

In terms of tolerability, some DAs had significantly fewer incidents of discontinuation due to all causes than some drugs of other drug classes, and safinamide showed a lower risk of discontinuation due to all causes than COMTIs. Ropinirole had the highest SUCRA value for tolerability, followed by pramipexole, safinamide, and ropinirole ER. In a previous NMA, pramipexole and ropinirole ER/immediate release were associated with a lower incidence of discontinuation due to all causes^13^. In contrast, opicapone, entacapone, and selegiline were less well-tolerated. Given that ropinirole and pramipexole ranked the highest in efficacy, while safinamide ranked the highest with the lowest risk of AE incidence (Fig. 4 and Supplementary Fig. 5A, D) in our NMA, the higher efficacy of ropinirole and pramipexole and better safety of safinamide may contribute to their greater tolerability.

This NMA also compared the incidence rates of 3 specific AEs: dyskinesia, hallucination, and OH. The ranking for low risk of incidence of dyskinesia was highest with selegiline, followed by rasagiline, istradefylline, and entacapone. Overall, these results were generally inversely correlated with the ranking of change in daily off-time, which is consistent with the fact that dyskinesia is caused by the excessive dopaminergic action of anti-PD drugs. Hallucination occurred more frequently in patients treated with some DAs than in those treated with other classes of anti-PD drugs. The 2 COMTIs showed different results, with entacapone ranking the highest with a lower incidence of hallucination, whereas opicapone ranked the second lowest. Vokurka et al. previously reported that hallucination was more frequent or intense after switching from entacapone to opicapone^65^. Several other reports have indicated a risk of hallucination associated with opicapone^66,67^. Opicapone should thus be used with caution, especially in patients with a history of or existing hallucination symptoms. The ranking for low incident rate of OH was the highest with rotigotine transdermal patch, followed by safinamide, pramipexole, and selegiline. However, the low frequency and the less understood pathology make it difficult to discuss these results in further detail. Regardless, it is important to evaluate the incidence of OH because it is an AE that often leads to discontinuation of therapy.

**Fig. 5:**
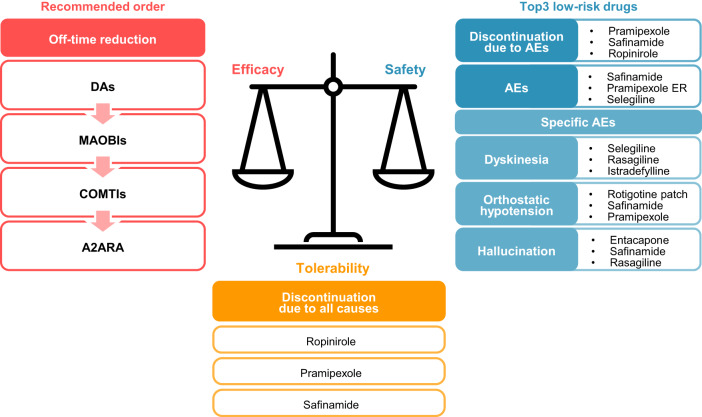
Summary of the results. Efficacy outcomes are listed by drug class in descending order of the effect size. For the tolerability outcome, drug names are listed in descending order of tolerability. For the safety outcomes, drug names are listed in ascending order of risk. All outcomes were evaluated based on the results of the RCTs, short-term use compared to clinical practice. DA dopamine agonist, MAOBI monoamine oxidase type B inhibitor, COMTI catechol-O-methyl transferase inhibitor, A2ARA adenosine A2A receptor antagonist, ER extended release, rotigotine *patch* rotigotine transdermal patch, AE adverse event, RCT randomized controlled trial.

Our NMA results are further summarized in Fig. 5. The DAs and MAOBIs were found to be more effective than the COMTIs and the A2ARA in terms of reducing the daily off time in patients with fluctuating PD. All the anti-PD drugs had different SUCRA-ranking profiles for each of the safety outcomes. The incidence of discontinuation due to all causes suggested that the efficacy and safety of ropinirole, pramipexole, and safinamide were better balanced than those of the other anti-PD drugs.

This study has some limitations. First, we only included RCTs. The duration of the included studies varied substantially from 3 weeks to 9 months, none of which was long enough to consider the long-term effects of anti-PD drugs. For example, impulse control disorder (ICD), which is thought to be one of the major reasons for the discontinuation of DAs^68^, often appears after long-term use and thus could not be evaluated in our study^69^. While pramipexole ER was found to be less effective than pramipexole in our study, ICD for pramipexole ER was reported to be significantly lower than that for pramipexole in a survey based on medical records and clinical interviews^70^. Therefore, pramipexole ER may be superior to pramipexole in terms of safety in clinical practice. Furthermore, study designs tend to differ according to the drug class. For example, dose-escalation models have been used in most studies on DAs but not in many studies on other drug classes (DAs: 20/20 studies MAOBIs: 3/13 studies, COMTIs: 0/14 studies, A2ARA: 0/9 studies). A dose escalation design may lead to fewer AEs. This study did not consider differences in study design, which may have affected the results. The levodopa equivalent daily doses (LEDDs) of drugs in RCTs differ between drug classes; therefore, these differences may affect efficacy. In addition, the total LEDD was not considered in the present NMA, although combination therapies with ≥2 anti-PD drugs were used in many RCTs. Second, not all drugs adjunctive to levodopa were analyzed in our NMA. For example, ergot DAs and amantadine were excluded from this study. Although ergot DAs are effective for patients with PD with wearing-off, few clinical trials have evaluated their off-time. In addition, considering the prescription patterns of ergot DAs^71,72^, we excluded these drugs from our analysis. Amantadine was not included in the present study because of the small number of RCTs for wearing-off^73,74^ and differences in study design and indications; it was mainly used for patients with dyskinesia. Recently, amantadine delayed release/ER was approved for dyskinesia and wearing-off^75^, and is useful for advanced PD. Third, the severity of AEs was not considered. Fourth, although no global inconsistency was confirmed in our NMA for the outcome of discontinuation due to all causes, there was an inconsistency between the direct and indirect evidence that compared entacapone with placebo for this outcome (Supplementary Table 3B). This may be caused by the inconsistent results obtained from some RCTs: one directly compared entacapone with placebo, while the other compared entacapone with opicapone, rasagiline, or istradefylline. The comparison pairs of the active treatments in the latter study were found only in this RCT, and therefore, the results from this RCT may have strongly affected the results of the present NMA, causing this inconsistency. Fifth, there were some heterogeneities in the study results comparing the same 2 anti-PD drugs (I-squared >50%) (Supplementary Table 2). This may also have been caused by differences in the study design among the studies (e.g., the duration of treatment, dose, study patients, etc.). Finally, the relative efficacy, tolerability, and safety of the medications are discussed based on the SUCRA values. Differences in SUCRA values depend on the analysis design, which makes it difficult to determine the clinical importance of a certain difference in SUCRA^76^. Further quantitative comparisons between drugs are provided by SMDs or ORs in the league tables of the NMA results^77^.

We compared and ranked the efficacy, tolerability, and safety of anti-PD drugs from different drug classes in this NMA and found that ropinirole, pramipexole, and safinamide were well-balanced anti-PD drugs that satisfy both change in daily off-time and discontinuation due to all causes. Furthermore, we evaluated 5 safety outcomes (discontinuation due to AEs and the incidence of AEs, dyskinesia, hallucination, and OH). We believe that our findings can be referred when physicians select the appropriate drug for each patient with fluctuating PD in clinical practice.

## Methods

### Search strategy

We followed the Preferred Reporting Items for Systematic Reviews and Meta-analyses for Network Meta-analyses (PRISMA-NMA) reporting guidelines^78^. The protocol was registered at PROSPERO (CRD42021270256) on August 27, 2021.

We systematically searched the PubMed, Embase, and Cochrane Library databases for articles published from their inception through July 21, 2021. Keywords included Parkinson disease, pramipexole, ropinirole, rotigotine, rasagiline, safinamide, selegiline, entacapone, opicapone, istradefylline, and randomized (Supplementary Table 4 shows a detailed list of the search terms). All titles and abstracts were independently screened by two reviewers, and potentially relevant articles were selected for full-text review. Full-text screenings were conducted independently by the same two reviewers, and any disagreements were resolved by consultation with a third reviewer.

### Inclusion and exclusion criteria

We included studies meeting the following criteria: (1) randomized controlled trials; (2) written in English; (3) included patients with PD and motor fluctuations or wearing-off who received levodopa; (4) included as outcomes at least one of the following endpoints: change in daily off-time, discontinuation due to all causes, discontinuation due to AEs, and incidences of AEs, dyskinesia, hallucination, and OH; (5) compared placebo with anti-PD drug(s) or among anti-PD drugs (selected anti-PD drugs: pramipexole, pramipexole ER, ropinirole, ropinirole ER, ropinirole transdermal patch, rotigotine transdermal patch, rasagiline, safinamide, selegiline, entacapone, opicapone, and istradefylline); (6) included study arms at approved dosage of selected anti-PD drugs in Japan, the USA, EU, or UK.

In addition, we excluded studies meeting the following criteria: (1) narrative reviews, systematic reviews, meta-analyses, meeting summaries; (2) no original data (previously reported data only).

In addition to the publications from the literature search, six eligible reports from CTD for drug application in Japan (available from the website of the Pharmaceuticals and Medical Devices Agency, which is a Japanese regulatory agency) of the targeted drugs were also included.

### Data extraction and quality assessment

Two researchers independently extracted the data. The extracted data included change in daily off-time (efficacy outcome); discontinuation due to all causes (tolerability outcome); and discontinuation due to AEs and the incidence of AEs, dyskinesia, hallucination/visual hallucination, and OH/postural hypotension (safety outcomes). For missing data of extracted articles, we additionally searched CTD for drug application in Japan and extracted eligible data. The following data were also extracted: authors’ names, publication year, country, comparison, study period, sample size, gender, age, and levodopa daily dose.

Two researchers independently assessed the quality of the included studies and classified each study as having a “low risk of bias,” “some concerns,” or “high risk of bias”^79,80^.

### Statistical analysis

The NMA was performed under a frequentist framework using a random-effects model with the “network” and “metan” packages in Stata (version 13.0; StataCorp, LLC)^81,82^. Network maps were then generated for each analysis. Summary results were presented as SMDs with 95% CIs for change in daily off-time and ORs with 95% CIs for the tolerability and safety outcomes. A 95% CI of an SMD not covering 0 or a 95% CI of an OR not covering 1 indicated a statistically significant association. For each outcome, the SUCRA was used to rank each drug separately^76^. A greater SUCRA value (range, 0% to 100%) indicates a higher ranking in efficacy, tolerability, and safety. We confirmed the assumption of consistency for the NMA with a global inconsistency test using a side-splitting approach to compare direct and indirect evidence. Inconsistency was defined as a difference (*P* < 0.05) between the direct and indirect evidence^83^. Using the data from direct comparisons, the heterogeneity test and I-squared values were also calculated for each drug comparison^84^.

### Supplementary information

Supplementary information

Related Manuscript File

## Data Availability

All data generated or analyzed during this study are included in this published article and its supplementary information files.
