# Supplementary material for: Comparative efficacy and safety of adjunctive drugs to levodopa for fluctuating Parkinson’s disease - network meta-analysis
Source: NPJ Parkinsons Dis. 2023 Oct 19;9:143. doi: 10.1038/s41531-023-00589-8 (PMC10584871; doi:10.1038/s41531-023-00589-8)
Supplement: Supplementary file 1 — Supplementary information [file 41531_2023_589_MOESM1_ESM.pdf]

Supplementary Information

Supplementary Table 1. Characteristics of the included studies
Supplementary Figure 1. Risk of bias assessment of the included studies from literature search
Supplementary Figure 2. Network maps for safety outcomes
Supplementary Figure 3. League tables of the NMA results
Supplementary Figure 4. Forest plots of the NMA results
Supplementary Table 2. Heterogeneity of each comparison
Supplementary Table 3. Inconsistency test result for direct and indirect comparison
Supplementary Figure 5. Surface under the cumulative ranking curve (SUCRA) results
Supplementary Table 4. Search strategies

Supplementary Table 1. Characteristics of the included studies

Source	Country	Comparison	Study period	Sample size, N	Male, %	Age, years, mean (SD)	Levodopa daily dose, mg, mean (SD)	Outcome events*
Barone P, 2007	25 countries	Placebo	26 weeks	314	61	65.1	618.1	a,b,c,d,e,f
		Ropinirole 0.75 up to 24 mg/day		310	63	64.1	663.1	
Borgohain R, 2014	India, Romania, Italy	Placebo	24 weeks	222	72.1	59.4 (9.41)	N/A	a,b,c,d,e
		Safinamide 50 mg/day		223	70.4	60.1 (9.65)	N/A	
		Safinamide 100 mg/day		224	72.8	60.1 (9.19)	N/A	
Brooks DJ, 2003	the United Kingdom, the Republic of Ireland	Placebo	6 months	57	70.2	64.7 (8.5)	712 (369)	d,e,f
		Entacapone 200 mg		115	60.0	65.9 (8.9)	682 (390)	
EUCTR2004-000817-20-IT	Argentina, Austria, Chile, Estonia, France, India, Italy, Latvia, Lithuania, Russia, Republic of South Africa, Spain, United Kingdom, Ukraine	Placebo	16 weeks	152	N/A	N/A	N/A	a,c,d,e
		Entacapone 200 mg		153	N/A	N/A	N/A	
		Istradefylline 40 mg/day		159	N/A	N/A	N/A	
EUCTR2013-002254-70-DE	the United States	Placebo	12 weeks	204	60.8	63.8	N/A	a,b,c,e,f,g
		Istradefylline 20 mg/day		202	61.9	63.6	N/A	
		Istradefylline 40 mg/day		207	60.9	64.5	N/A	
Fenelon G, 2003	France and Spain	Placebo	3 months	63	60	65.0 (6.61)	N/A	a,b,c,d,e,f
		Entacapone 200 mg		99	64	63.5 (9.96)	N/A	

Source	Country	Comparison	Study period	Sample size, N	Male, %	Age, years, mean (SD)	Levodopa daily dose, mg, mean (SD)	Outcome events*
Ferreira JJ, 2015	Romania and Ukraine	Placebo	3-4 weeks	10	50	67.8 (7.97)	N/A	a,c
		Opicapone 5 mg/day		10	40	68.8 (7.60)	N/A	
		Opicapone 15 mg/day		10	60	63.5 (9.56)	N/A	
		Opicapone 30 mg/day		10	50	70.0 (10.72)	N/A	
Ferreira JJ, 2016	19 European countries and Russia	Placebo	14-15 weeks	121	59	64.3 (9.3)	675 (302)	a,b,c,d,e,f
		Opicapone 5 mg/day		122	58	63.6 (9.3)	642 (310)	
		Opicapone 25 mg/day		119	56	64.4 (9.0)	654 (324)	
		Opicapone 50 mg/day		115	60	63.5(9.2)	695 (338)	
		Entacapone 200 mg		122	62	63.7 (8.8)	645 (323)	
Golbe LI, 1988	the United States	Placebo	6 weeks	46	N/A	63.4	N/A	e,f
		Selegiline 10 mg/day		50	N/A	61.4	N/A	
Guttman M, 1997	6 European countries and Canada	Placebo	9 months 11 days (max)	83	63.9	63.72 (10.35)	N/A	c,d,e,f,g
		Pramipexole 0.375-4.5 mg/day		80	60.8	62.89 (10.03)	N/A	
Hattori N, 2018	Japan	Placebo	26 weeks	141	37.6	66.3 (7.62)	399.3 (141.03)	a,b,c,d,e,f
		Rasagiline 0.5 mg/day		134	43.3	66.1 (8.74)	407.8 (134.15)	
		Rasagiline 1 mg/day		129	35.7	65.8 (8.48)	420.7 (166.42)	
Hattori N, 2020a	Japan	Placebo	24 weeks	141	41.9	68.64 (7.66)	420.04 (123.90)	a,b,c,d,e
		Safinamide 50 mg/day		133	44.3	67.18 (9.04)	445.61 (156.66)	
		Safinamide 100 mg/day		132	49.2	68.36 (9.04)	446.48 (153.49)	
Hattori N, 2020b	Japan	Placebo	16 weeks	35	N/A	N/A	N/A	a
		Ropinirole patch up to 64 mg/day		71	N/A	N/A	N/A	
		Ropinirole ER up to 16 mg/day		71	N/A	N/A	N/A	

Source	Country	Comparison	Study period	Sample size, N	Male, %	Age, years, mean (SD)	Levodopa daily dose, mg, mean (SD)	Outcome events*
Hauser RA, 2008	the United Kingdom	Placebo	12 weeks	115	67.0	64 (10.2)	631 (356.7)	a,b,c,d,e
		Istradefylline 20 mg/day		115	66.1	63 (9.5)	652 (370.6)	
Hauser RA, 2015	Eastern Europe, the European Union, India, Latin America, North America, Turkey	Placebo	12 weeks	155	50.3	63.0 (8.4)	median 650 (range 50 - 3600)	a,b,c,d,e
		Rasagiline 1 mg/day		154	61.7	63.6 (9.0)	median 800 (range 100 - 2500)	
Lees AJ, 2017	Belgium, UK, Israel, Estonia, Czech Republic, Russia, South Africa, Australia, South Korea, India, Argentina, Chile	Placebo	14-15 weeks	136	52.6	61.5 (8.9)	714 (338)	a,b,c,d,e
		Opicapone 25 mg/day		125	65.6	62.5 (8.5)	806 (398)	
		Opicapone 50 mg/day		150	60.5	65.5 (8.4)	700 (312)	
LeWitt PA, 2007	the United States and Canada	Placebo	24 weeks	120	N/A	66.3 (9.6)	753 (470)	a,e,f,g
		Rotigotine patch 4-8 mg/day		118	N/A	66.5 (10.0)	760 (601)	
		Rotigotine patch 4-12 mg/day		111	N/A	64.5 (10.4)	740 (407)	
LeWitt PA, 2008	US and Canada	Placebo	12 weeks	66	60.6	64 (10)	589 (301)	a,b,c,d
		Istradefylline 40 mg/day		129	59.7	63 (9)	560 (291)	
Lieberman A, 1997	the United States, Canada	Placebo	32 weeks	179	65	63.3	819.19 (466.08)	b,c,f
		Pramipexole from 0.375 to 4.5 mg/day		181	66	63.4	843.37 (578.86)	
Lieberman A, 1998	the United States	Placebo	24 weeks	54	N/A	N/A	843 (517)	b,c,e,g
		Ropinirole 0.75 to 24 mg/day		95	N/A	N/A	759 (422)	

Source	Country	Comparison	Study period	Sample size, N	Male, %	Age, years, mean (SD)	Levodopa daily dose, mg, mean (SD)	Outcome events*
Mizuno Y, 2007a	Japan	Placebo	8 weeks	113	N/A	62.7 (9.9)	431.1 (130.7)	a,b,c,d,e
		Entacapone 100 mg		113	N/A	62.9 (8.9)	431.1 (132.3)	
		Entacapone 200 mg		114	N/A	62.7 (8.7)	455.1 (161.0)	
Mizuno Y, 2007b	Japan	Placebo	16 weeks	63	N/A	N/A	N/A	a
		Ropinirole 0.75 to 15.0 mg/day		64	N/A	N/A	N/A	
Mizuno Y, 2010	Japan	Placebo	12 weeks	119	38.1	65.0 (7.6)	426.3 (143.0)	a,b,c,d,e,f
		Istradefylline 20 mg/day		118	43.5	65.1 (7.2)	407.0 (113.1)	
		Istradefylline 40 mg/day		125	44.4	63.7 (8.6)	415.3 (159.2)	
Mizuno Y, 2013	Japan	Placebo	12 weeks	126	47.2	65.8 (8.6)	425.4 (146.4)	a,b,c,d,e,f,g
		Istradefylline 20 mg/day		123	33.3	66.1 (8.6)	430.8 (156.5)	
		Istradefylline 40 mg/day		124	52.0	65.7 (9.0)	420.5 (131.8)	
Mizuno Y, 2014	Japan	Placebo	16 weeks	57	N/A	N/A	N/A	a
		Rotigotine patch 2-16 mg/day		111	N/A	N/A	N/A	
		Ropinirole 0.75-15 mg/day		113	N/A	N/A	N/A	
NCT01268891	Korea	Placebo	18 weeks	66	65.2	59.5 (9.0)	N/A	a,b,c,d,e
		Rasagiline 1 mg/day		66	50.0	60.2 (8.6)	N/A	
Nicholas AP, 2014	US	Placebo	16 weeks	108	69	64.8 (10.2)	642.8 (420.3)	a,b,c,d,e,f,g
		Rotigotine patch 2 mg/day		101	76	65.4 (10.5)	643.3 (344.5)	a,b,c,d,e,g
		Rotigotine patch 4 mg/day		107	74	64.6 (9.0)	627.7 (359.4)	
		Rotigotine patch 6 mg/day		104	70	64.6 (10.4)	619.0 (376.4)	
		Rotigotine patch 8 mg/day		94	60	63.2 (11.6)	643.0 (365.8)	
		Rotigotine patch overall (2-8 mg/day)		406	N/A	N/A	N/A	b,c,d,e,f,g
Nomoto M, 2014	Japan	Placebo	12 weeks	56	N/A	N/A	N/A	a
		Rotigotine patch 2-16 mg/day		54	N/A	N/A	N/A	

Source	Country	Comparison	Study period	Sample size, N	Male, %	Age, years, mean (SD)	Levodopa daily dose, mg, mean (SD)	Outcome events*
Ondo WG, 2007	the United States	Placebo	12 weeks	50	72.0	66.3 (10.6)	N/A	a,b,c,d,e
		Selegiline ODT 2.5 mg/day		100	69.4	68.4 (9.0)	N/A	
Pahwa R, 2007	Belgium, the Czech Republic, France, Hungary, Italy, Poland, Spain, and the United States	Placebo	24 weeks	191	68	66.0 (9.7)	776 (357.3)	a,b,c,d,e,f,g
		Ropinirole PR 2-24mg/day		202	58	66.3 (9.2)	824 (424.4)	
Parkinson Study Group, 2005	N/A	Placebo	26 weeks	159	65.4	64.5 (9.9)	821 (485)	a,b
		Rasagiline 0.5 mg/day		164	62.2	62.6 (9.5)	750 (379)	
		Rasagiline 1.0 mg/day		149	66.4	62.9 (8.9)	815 (471)	
Poewe WH, 2002	Germany and Austria	Placebo	24 weeks	88	N/A	N/A	N/A	a
		Entacapone 200 mg		172	N/A	N/A	N/A	
Poewe WH, 2007	Europe, South Africa, Australia, and New Zealand	Placebo	16 weeks	101	71	65.0 (10.0)	814 (398)	a,b,c,d,e,f,g
		Pramipexole 0.375-4.5mg/day		201	56	63.2 (9.7)	813 (459)	
		Rotigotine patch 4-16mg/day		204	66	64.3 (9.0)	795 (380)	
Pourcher E, 2012	US and Canada	Placebo	12 weeks	151	64.2	63 (8.3)	N/A	a,b,c,d,e,f
		Istradefylline 10 mg/day		153	67.3	63 (8.9)	N/A	
		Istradefylline 20 mg/day		149	69.1	64 (9.8)	N/A	
		Istradefylline 40 mg/day		152	65.8	63 (9.3)	N/A	

Source	Country	Comparison	Study period	Sample size, N	Male, %	Age, years, mean (SD)	Levodopa daily dose, mg, mean (SD)	Outcome events*
Rascol O, 1996	France, England	Placebo	12 weeks	23	60.9	63 (9)	715 (353)	b,c,d,e,g
		Ropinirole 1-8.0 mg/day		23	60.9	62 (7)	663 (302)	
Rascol O, 2005	Europe, Israel, and Argentina	Placebo	18 weeks	229	58	64.8 (8.8)	697 (295)	a,b,c,d,e,f,g
		Rasagiline 1 mg/day		231	67	63.9 (9.0)	722 (334)	
		Entacapone 200 mg		227	61	63.0 (9.4)	706 (321)	
Rascol O, 2012	20 countries	Placebo	18 weeks	247	60	63.6 (8.82)	N/A	a,b,c
		Entacapone 200 mg		234	58	63.7 (9.88)	N/A	
Reichmann H, 2005	N/A	Placebo	13 weeks	96	59	66 (9)	533 (231)	a,b,c,d,e
		Entacapone 200 mg		174	54	67 (8)	566 (243)	
Schapira A, 2011	Austria, Czech Republic, Hungary, India, Italy, Philippines, Poland, Russia, Slovakia, South Korea, Spain, Sweden, Ukraine, and the United Kingdom	Placebo	18 weeks	178	52.8	60.9 (9.7)	569.3	a,b,c,d,e,f,g
		Pramipexole ER 0.375-4.5 mg/day		164	56.1	61.6 (9.7)	568.0	
		Pramipexole IR 0.375-4.5 mg/day		175	56.0	62.0 (10.3)	609.7	
Schapira A, 2017	21 countries in Europe, the Asia-Pacific region, and North America	Placebo	24 weeks	275	59.3	62.1 (8.9)	792.3 (400.7)	a,b,c,d,e,f
		Safinamide 50-100 mg/day		274	62.4	61.7 (9.0)	760.8 (445.9)	

Source	Country	Comparison	Study period	Sample size, N	Male, %	Age, years, mean (SD)	Levodopa daily dose, mg, mean (SD)	Outcome events*
Stacy M, 2008	North America	Placebo	12 weeks	77	70.1	63.0 (12.05)	N/A	a,b,c
		Istradefylline up to 20mg		163	63.8	65.0 (9.59)	N/A	
		Istradefylline up to 60mg		155	68.4	63.5 (10.08)	N/A	
Takeda A, 2021	Japan	Placebo	14-15 weeks	147	38.1	68.5 (8.6)	422.3 (170.1)	a,b,c,d,e
		Opicapone 25 mg/day		145	40.0	67.9 (9.1)	407.9 (147.0)	
		Opicapone 50 mg/day		145	41.4	67.4 (7.8)	445.3 (175.8)	
Waters CH, 2004	the United States	Placebo	12 weeks	46	65	64 (11.1)	N/A	b,c
		Selegiline ODT 1.25-2.5 mg/day		94	63	66 (9.3)	N/A	
		Placebo (Week 4-6)		46	65	64 (11.1)	N/A	a
		Selegiline ODT 1.25 mg/day (Week 4-6)		94	63	66 (9.3)	N/A	
		Placebo (Week 10-12)		46	65	64 (11.1)	N/A	a
		Selegiline ODT 2.5 mg/day (Week 10-12)		94	63	66 (9.3)	N/A	
Zesiewicz TA, 2017	Argentina, Chile, Estonia, the Russian Federation, Slovakia, the Republic of Korea and the USA	Placebo	4 weeks	75	45	63.8 (10.02)	N/A	a,b,c,d,e
		Ropinirole PR 4 mg/day		25	52	66.5 (7.45)	N/A	
		Ropinirole PR 8 mg/day		76	57	65.6 (9.19)	N/A	
		Ropinirole PR 12 mg/day		75	56	65.2 (9.62)	N/A	
		Ropinirole PR 16 mg/day		76	51	63.8 (9.15)	N/A	
		Ropinirole PR 24 mg/day		25	60	66.9 (7.94)	N/A	
		Ropinirole PR overall (4-24 mg/day)		276	N/A	N/A	N/A	d,e
Zhang L, 2013	China	Placebo	12 weeks	125	53.6	61.56 (9.50)	521	a,b,c,d,e
		Rasagiline 1 mg/day		119	53.78	61.64 (8.53)	515.2	

Source	Country	Comparison	Study period	Sample size, N	Male, %	Age, years, mean (SD)	Levodopa daily dose, mg, mean (SD)	Outcome events*
Zhang Z, 2013	China	Placebo	24 weeks	170	61.8	63.6 (10.5)	539.8 (426.2)	a,b,c,d,e,f,g
		Ropinirole PR 2 to 24 mg/day		175	66.3	64.1 (9.0)	506.6 (290.9)	
Zhang Z, 2018	China	Placebo	16 weeks	158	69	61.7 (9.9)	550 (224)	a,b,c,d,e,f
		Rasagiline 1 mg/day		163	63	62.7 (8.9)	501 (222)	
Zhang ZX, 2017	China	Placebo	19 weeks	172	64.0	62.8 (9.1)	652.44 (330.81)	a,b,c,d,e
		Rotigotine patch 4 mg to 16 mg/day		174	53.4	61.7 (8.8)	593.92 (295.16)	

*a represents change in daily off-time; b, discontinuation due to all causes; c, discontinuation due to AEs; d, AEs; e, dyskinesia; f, hallucination; g, orthostatic hypotension.

Abbreviations: SD, standard deviation; rotigotine patch, rotigotine transdermal patch; ropinirole patch, ropinirole transdermal patch; ODT, orally disintegrating tablet; PR, prolonged release; ER, extended release; IR, immediate release; AE, adverse event.

	Randomisation process	Deviations from the intended interventions	Missing outcome data	Measurement of the outcome	Selection of the reported result
Barone P, 2007	low	low	low	some concerns	low
Borgohain R, 2014	low	low	low	some concerns	low
Brooks DJ, 2003	low	low	high	low	low
EUCTR2004-000817-20-IT	some concerns	low	low	low	low
EUCTR2013-002254-70-DE	some concerns	low	some concerns	low	low
Fenelon G, 2003	some concerns	low	low	low	low
Ferreira JJ, 2015	some concerns	low	some concerns	low	low
Ferreira JJ, 2016	low	low	low	low	low
Golbe LI, 1988	some concerns	low	some concerns	low	low
Guttman M, 1997	some concerns	low	low	low	low
Hattori N, 2018	some concerns	low	low	low	low
Hattori N, 2020a	some concerns	low	low	low	low
Hattori N, 2020b	low	low	low	low	low
Hauser RA, 2008	some concerns	low	low	low	low
Hauser RA, 2015	low	low	some concerns	low	low
Lees AJ, 2017	some concerns	low	low	low	low
LeWitt PA, 2007	low	low	low	low	low
LeWitt PA, 2008	some concerns	low	low	low	low
Lieberman A, 1997	low	low	low	low	low
Lieberman A, 1998	low	low	low	low	low
Mizuno Y, 2007a	some concerns	low	some concerns	low	low
Mizuno Y, 2007b	some concerns	low	high	some concerns	some concerns
Mizuno Y, 2010	some concerns	low	low	low	low
Mizuno Y, 2013	some concerns	low	some concerns	low	low
Mizuno Y, 2014	low	low	some concerns	low	low

**NCT01268891**	some concerns	low	some concerns	low	low
**Nicholas AP, 2014**	low	low	low	low	low
**Nomoto M, 2014**	low	low	low	low	low
**Ondo WG, 2007**	low	low	low	low	low
**Pahwa R, 2007**	low	low	low	low	low
**Parkinson Study Group, 2005**	low	low	low	low	low
**Poewe WH, 2002**	low	low	low	low	low
**Poewe WH, 2007**	low	low	low	low	low
**Pourcher E, 2012**	low	low	low	low	low
**Rascol O, 1996**	some concerns	low	low	low	low
**Rascol O, 2005**	low	low	low	low	low
**Rascol O, 2012**	some concerns	low	low	low	low
**Reichmann H, 2005**	some concerns	low	low	low	low
**Schapira A, 2011**	low	low	low	low	low
**Schapira A, 2017**	low	low	low	low	low
**Stacy M, 2008**	some concerns	low	low	low	low
**Takeda A, 2021**	low	low	low	low	low
**Waters CH, 2004**	some concerns	low	low	low	low
**Zesiewicz TA, 2017**	low	low	low	low	low
**Zhang L, 2013**	low	some concerns	low	low	low
**Zhang Z, 2013**	some concerns	some concerns	low	low	low
**Zhang Z, 2018**	low	low	some concerns	low	low
**Zhang ZX, 2017**	low	low	low	low	low

Supplementary Figure 1. Risk of bias assessment of the included studies from literature search

The quality of the 48 studies included from literature search was assessed based on the Cochrane Handbook for Systematic Reviews of Interventions version 6.3, Chapter 8 and the revised tool for assessing risk of bias in randomized trials (Rob2), and each study was classified into having “low risk of bias,” “some concerns,” or “high risk of bias”.

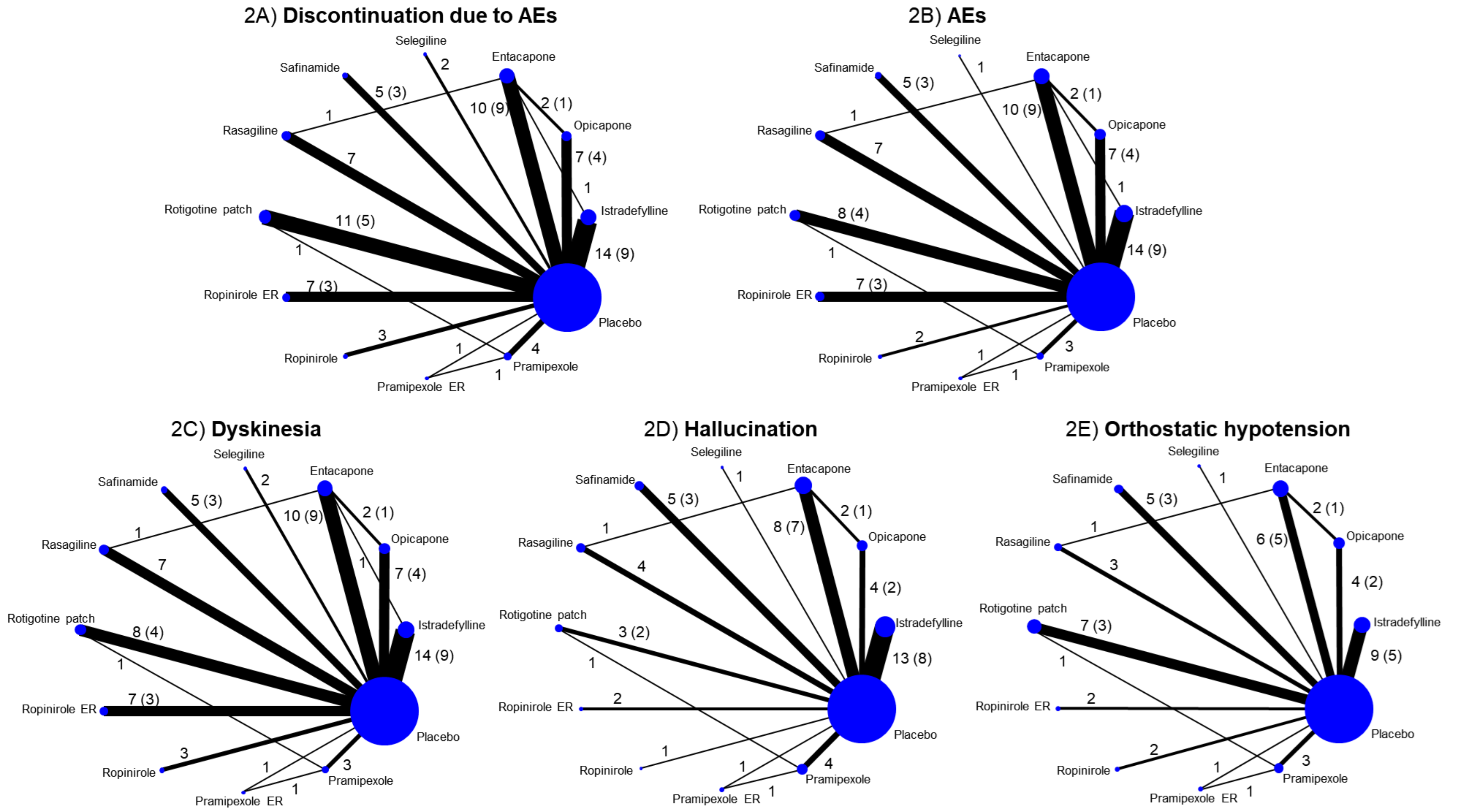

Supplementary Figure 2. Network maps for safety outcomes

The circular nodes indicate each treatment. The size of the nodes corresponds to the number of patients assigned to each treatment. Treatments with direct comparisons are linked with a line, and the thickness of the line corresponds to the number of comparisons. The figure next to the line shows the number of comparisons and the figure in parentheses shows the number of trials if there are differences between the number of comparisons and trials.

Abbreviations: AE, adverse event; ER, extended release; ropinirole patch, ropinirole transdermal patch; rotigotine patch, rotigotine transdermal patch.

3A) Change in daily off-time

**PBO**	**-0.41 (-0.56,-0.25)**	-0.18 (-0.40,0.03)	**-0.35 (-0.50,-0.21)**	**-0.40 (-0.52,-0.28)**	**-0.48 (-0.73,-0.24)**	**-0.29 (-0.38,-0.20)**	**-0.35 (-0.45,-0.24)**	**-0.38 (-0.50,-0.25)**	**-0.37 (-0.66,-0.09)**	**-0.29 (-0.37,-0.21)**	**-0.30 (-0.41,-0.19)**	**-0.18 (-0.27,-0.10)**
**0.41 (0.25,0.56)**	**PPX**	**0.23 (0.01,0.44)**	0.05 (-0.16,0.27)	0.01 (-0.19,0.21)	-0.08 (-0.37,0.21)	0.12 (-0.05,0.28)	0.06 (-0.13,0.25)	0.03 (-0.17,0.23)	0.03 (-0.29,0.36)	0.12 (-0.06,0.29)	0.11 (-0.08,0.30)	**0.23 (0.05,0.40)**
0.18 (-0.03,0.40)	**-0.23 (-0.44,-0.01)**	**PPX ER**	-0.17 (-0.43,0.09)	-0.21 (-0.46,0.03)	-0.30 (-0.63,0.03)	-0.11 (-0.34,0.12)	-0.16 (-0.40,0.08)	-0.20 (-0.45,0.05)	-0.19 (-0.55,0.16)	-0.11 (-0.34,0.12)	-0.12 (-0.36,0.13)	0.00 (-0.23,0.23)
**0.35 (0.21,0.50)**	-0.05 (-0.27,0.16)	0.17 (-0.09,0.43)	**ROP**	-0.04 (-0.22,0.14)	-0.13 (-0.41,0.15)	0.06 (-0.10,0.23)	0.01 (-0.18,0.19)	-0.03 (-0.22,0.17)	-0.02 (-0.34,0.30)	0.06 (-0.11,0.23)	0.06 (-0.13,0.24)	**0.17 (0.00,0.34)**
**0.40 (0.28,0.52)**	-0.01 (-0.21,0.19)	0.21 (-0.03,0.46)	0.04 (-0.14,0.22)	**ROP ER**	-0.09 (-0.34,0.16)	0.11 (-0.04,0.26)	0.05 (-0.11,0.21)	0.02 (-0.15,0.19)	0.02 (-0.28,0.33)	0.11 (-0.04,0.25)	0.10 (-0.06,0.26)	**0.22 (0.07,0.36)**
**0.48 (0.24,0.73)**	0.08 (-0.21,0.37)	0.30 (-0.03,0.63)	0.13 (-0.15,0.41)	0.09 (-0.16,0.34)	**ROP patch**	0.19 (-0.07,0.45)	0.14 (-0.13,0.40)	0.11 (-0.17,0.38)	0.11 (-0.26,0.48)	0.19 (-0.06,0.45)	0.19 (-0.08,0.45)	**0.30 (0.04,0.56)**
**0.29 (0.20,0.38)**	-0.12 (-0.28,0.05)	0.11 (-0.12,0.34)	-0.06 (-0.23,0.10)	-0.11 (-0.26,0.04)	-0.19 (-0.45,0.07)	**RTG patch**	-0.06 (-0.19,0.08)	-0.09 (-0.24,0.06)	-0.08 (-0.38,0.21)	-0.00 (-0.12,0.12)	-0.01 (-0.15,0.13)	0.11 (-0.01,0.23)
**0.35 (0.24,0.45)**	-0.06 (-0.25,0.13)	0.16 (-0.08,0.40)	-0.01 (-0.19,0.18)	-0.05 (-0.21,0.11)	-0.14 (-0.40,0.13)	0.06 (-0.08,0.19)	**RAS**	-0.03 (-0.20,0.13)	-0.03 (-0.33,0.27)	0.06 (-0.07,0.18)	0.05 (-0.10,0.20)	**0.16 (0.03,0.30)**
**0.38 (0.25,0.50)**	-0.03 (-0.23,0.17)	0.20 (-0.05,0.45)	0.03 (-0.17,0.22)	-0.02 (-0.19,0.15)	-0.11 (-0.38,0.17)	0.09 (-0.06,0.24)	0.03 (-0.13,0.20)	**SAF**	0.01 (-0.30,0.31)	0.09 (-0.06,0.24)	0.08 (-0.09,0.25)	**0.20 (0.05,0.35)**
**0.37 (0.09,0.66)**	-0.03 (-0.36,0.29)	0.19 (-0.16,0.55)	0.02 (-0.30,0.34)	-0.02 (-0.33,0.28)	-0.11 (-0.48,0.26)	0.08 (-0.21,0.38)	0.03 (-0.27,0.33)	-0.01 (-0.31,0.30)	**SEL**	0.08 (-0.21,0.38)	0.08 (-0.23,0.38)	0.19 (-0.10,0.48)
**0.29 (0.21,0.37)**	-0.12 (-0.29,0.06)	0.11 (-0.12,0.34)	-0.06 (-0.23,0.11)	-0.11 (-0.25,0.04)	-0.19 (-0.45,0.06)	0.00 (-0.12,0.12)	-0.06 (-0.18,0.07)	-0.09 (-0.24,0.06)	-0.08 (-0.38,0.21)	**ENT**	-0.01 (-0.13,0.12)	0.11 (-0.00,0.22)
**0.30 (0.19,0.41)**	-0.11 (-0.30,0.08)	0.12 (-0.13,0.36)	-0.06 (-0.24,0.13)	-0.10 (-0.26,0.06)	-0.19 (-0.45,0.08)	0.01 (-0.13,0.15)	-0.05 (-0.20,0.10)	-0.08 (-0.25,0.09)	-0.08 (-0.38,0.23)	0.01 (-0.12,0.13)	**OPC**	0.12 (-0.02,0.25)
**0.18 (0.10,0.27)**	**-0.23 (-0.40,-0.05)**	0.00 (-0.23,0.23)	**-0.17 (-0.34,-0.00)**	**-0.22 (-0.36,-0.07)**	**-0.30 (-0.56,-0.04)**	-0.11 (-0.23,0.01)	**-0.16 (-0.30,-0.03)**	**-0.20 (-0.35,-0.05)**	-0.19 (-0.48,0.10)	-0.11 (-0.22,0.00)	-0.12 (-0.25,0.02)	**ISD**

Global inconsistency test: Chi-square=3.04, P=0.932

SMD (95%CI)

3B) Discontinuation due to all causes

**PBO**	**0.62 (0.44,0.86)**	0.99 (0.52,1.87)	**0.49 (0.31,0.77)**	0.73 (0.51,1.06)	0.79 (0.63,1.01)	0.95 (0.71,1.28)	0.73 (0.52,1.02)	1.47 (0.51,4.20)	1.12 (0.89,1.41)	1.21 (0.85,1.70)	0.97 (0.76,1.24)
**1.62 (1.17,2.26)**	**PPX**	1.60 (0.84,3.05)	0.79 (0.45,1.39)	1.19 (0.72,1.95)	1.29 (0.88,1.88)	1.54 (0.99,2.40)	1.18 (0.74,1.90)	2.38 (0.79,7.16)	**1.82 (1.22,2.71)**	**1.95 (1.21,3.15)**	**1.57 (1.04,2.37)**
1.01 (0.54,1.92)	0.63 (0.33,1.19)	**PPX ER**	0.50 (0.23,1.09)	0.74 (0.36,1.55)	0.81 (0.41,1.58)	0.96 (0.48,1.94)	0.74 (0.36,1.52)	1.49 (0.43,5.08)	1.14 (0.58,2.23)	1.22 (0.59,2.52)	0.98 (0.50,1.95)
**2.04 (1.29,3.22)**	1.26 (0.72,2.21)	2.01 (0.92,4.41)	**ROP**	1.50 (0.83,2.71)	1.62 (0.97,2.71)	**1.94 (1.12,3.34)**	1.49 (0.85,2.63)	2.99 (0.95,9.43)	**2.29 (1.37,3.80)**	**2.46 (1.39,4.36)**	**1.98 (1.18,3.32)**
1.36 (0.94,1.97)	0.84 (0.51,1.38)	1.34 (0.64,2.81)	0.67 (0.37,1.21)	**ROP ER**	1.08 (0.70,1.68)	1.29 (0.81,2.07)	0.99 (0.60,1.64)	2.00 (0.66,6.08)	1.53 (0.99,2.35)	1.64 (0.99,2.72)	1.32 (0.85,2.06)
1.26 (0.99,1.59)	0.78 (0.53,1.13)	1.24 (0.63,2.43)	0.62 (0.37,1.03)	0.92 (0.60,1.43)	**RTG patch**	1.19 (0.82,1.74)	0.92 (0.61,1.39)	1.85 (0.63,5.42)	**1.41 (1.01,1.96)**	1.52 (1.00,2.31)	1.22 (0.87,1.72)
1.05 (0.78,1.42)	0.65 (0.42,1.01)	1.04 (0.51,2.10)	**0.52 (0.30,0.89)**	0.77 (0.48,1.24)	0.84 (0.57,1.22)	**RAS**	0.77 (0.49,1.21)	1.54 (0.52,4.60)	1.18 (0.83,1.67)	1.27 (0.81,1.99)	1.02 (0.70,1.50)
1.37 (0.98,1.92)	0.84 (0.53,1.36)	1.35 (0.66,2.78)	0.67 (0.38,1.18)	1.01 (0.61,1.66)	1.09 (0.72,1.65)	1.30 (0.83,2.04)	**SAF**	2.01 (0.67,6.07)	**1.53 (1.02,2.31)**	**1.65 (1.02,2.68)**	1.33 (0.87,2.02)
0.68 (0.24,1.95)	0.42 (0.14,1.27)	0.67 (0.20,2.30)	0.33 (0.11,1.05)	0.50 (0.16,1.52)	0.54 (0.18,1.59)	0.65 (0.22,1.93)	0.50 (0.16,1.50)	**SEL**	0.76 (0.26,2.24)	0.82 (0.27,2.49)	0.66 (0.22,1.95)
0.89 (0.71,1.12)	**0.55 (0.37,0.82)**	0.88 (0.45,1.73)	**0.44 (0.26,0.73)**	0.66 (0.43,1.01)	**0.71 (0.51,0.99)**	0.85 (0.60,1.20)	**0.65 (0.43,0.98)**	1.31 (0.45,3.84)	**ENT**	1.08 (0.74,1.57)	0.87 (0.63,1.20)
0.83 (0.59,1.17)	**0.51 (0.32,0.83)**	0.82 (0.40,1.69)	**0.41 (0.23,0.72)**	0.61 (0.37,1.01)	0.66 (0.43,1.00)	0.79 (0.50,1.23)	**0.61 (0.37,0.98)**	1.22 (0.40,3.68)	0.93 (0.63,1.36)	**OPC**	0.80 (0.53,1.23)
1.03 (0.81,1.32)	**0.64 (0.42,0.96)**	1.02 (0.51,2.01)	**0.51 (0.30,0.85)**	0.76 (0.49,1.18)	0.82 (0.58,1.15)	0.98 (0.67,1.44)	0.75 (0.50,1.14)	1.51 (0.51,4.45)	1.15 (0.84,1.59)	1.24 (0.82,1.90)	**ISD**

Global inconsistency test: Chi-square=9.04, P=0.107

OR (95%CI)

3C) Discontinuation due to AEs

**PBO**	0.81 (0.56,1.17)	1.04 (0.48,2.25)	0.91 (0.56,1.47)	1.07 (0.66,1.72)	0.97 (0.74,1.29)	1.09 (0.76,1.56)	0.92 (0.60,1.40)	2.87 (0.47,17.47)	1.26 (0.97,1.65)	1.19 (0.80,1.77)	1.25 (0.93,1.68)
1.24 (0.86,1.79)	**PPX**	1.28 (0.59,2.77)	1.13 (0.61,2.07)	1.32 (0.72,2.40)	1.21 (0.78,1.85)	1.34 (0.80,2.26)	1.13 (0.65,1.99)	3.55 (0.56,22.42)	1.56 (0.99,2.46)	1.47 (0.85,2.53)	1.54 (0.96,2.47)
0.97 (0.45,2.10)	0.78 (0.36,1.69)	**PPX ER**	0.88 (0.35,2.19)	1.03 (0.42,2.55)	0.94 (0.42,2.13)	1.05 (0.45,2.47)	0.89 (0.37,2.14)	2.77 (0.39,19.78)	1.22 (0.54,2.77)	1.15 (0.48,2.74)	1.20 (0.52,2.76)
1.10 (0.68,1.77)	0.89 (0.48,1.63)	1.14 (0.46,2.83)	**ROP**	1.17 (0.60,2.30)	1.07 (0.61,1.87)	1.19 (0.65,2.17)	1.01 (0.53,1.91)	3.15 (0.49,20.42)	1.39 (0.80,2.40)	1.30 (0.70,2.43)	1.37 (0.78,2.40)
0.94 (0.58,1.51)	0.76 (0.42,1.38)	0.97 (0.39,2.41)	0.85 (0.43,1.68)	**ROP ER**	0.91 (0.53,1.59)	1.02 (0.56,1.85)	0.86 (0.45,1.63)	2.69 (0.42,17.42)	1.18 (0.69,2.05)	1.11 (0.60,2.07)	1.17 (0.67,2.05)
1.03 (0.78,1.36)	0.83 (0.54,1.28)	1.06 (0.47,2.40)	0.93 (0.54,1.63)	1.09 (0.63,1.90)	**RTG patch**	1.11 (0.70,1.76)	0.94 (0.57,1.56)	2.94 (0.47,18.32)	1.30 (0.88,1.91)	1.22 (0.75,1.99)	1.28 (0.85,1.92)
0.92 (0.64,1.32)	0.74 (0.44,1.25)	0.95 (0.41,2.24)	0.84 (0.46,1.53)	0.98 (0.54,1.79)	0.90 (0.57,1.42)	**RAS**	0.84 (0.48,1.48)	2.64 (0.42,16.68)	1.16 (0.76,1.79)	1.09 (0.64,1.87)	1.15 (0.72,1.83)
1.09 (0.71,1.67)	0.88 (0.50,1.55)	1.13 (0.47,2.73)	0.99 (0.52,1.88)	1.16 (0.61,2.20)	1.06 (0.64,1.77)	1.18 (0.68,2.07)	**SAF**	3.13 (0.49,20.01)	1.38 (0.83,2.28)	1.30 (0.72,2.32)	1.36 (0.81,2.28)
0.35 (0.06,2.12)	0.28 (0.04,1.78)	0.36 (0.05,2.58)	0.32 (0.05,2.06)	0.37 (0.06,2.41)	0.34 (0.05,2.11)	0.38 (0.06,2.39)	0.32 (0.05,2.04)	**SEL**	0.44 (0.07,2.73)	0.41 (0.07,2.63)	0.43 (0.07,2.71)
0.79 (0.61,1.04)	0.64 (0.41,1.01)	0.82 (0.36,1.86)	0.72 (0.42,1.25)	0.84 (0.49,1.46)	0.77 (0.52,1.14)	0.86 (0.56,1.32)	0.73 (0.44,1.20)	2.27 (0.37,14.11)	**ENT**	0.94 (0.60,1.46)	0.99 (0.67,1.45)
0.84 (0.56,1.26)	0.68 (0.40,1.17)	0.87 (0.36,2.08)	0.77 (0.41,1.43)	0.90 (0.48,1.67)	0.82 (0.50,1.34)	0.91 (0.54,1.56)	0.77 (0.43,1.38)	2.42 (0.38,15.36)	1.06 (0.68,1.66)	**OPC**	1.05 (0.64,1.72)
0.80 (0.60,1.08)	0.65 (0.40,1.04)	0.83 (0.36,1.91)	0.73 (0.42,1.28)	0.86 (0.49,1.50)	0.78 (0.52,1.18)	0.87 (0.55,1.39)	0.74 (0.44,1.23)	2.30 (0.37,14.37)	1.01 (0.69,1.49)	0.95 (0.58,1.56)	**ISD**

Global inconsistency test: Chi-square=6.21, P=0.287 OR (95%CI)

3D) AEs

**PBO**	**1.35 (1.06,1.72)**	0.95 (0.68,1.31)	1.25 (0.90,1.74)	**1.41 (1.13,1.76)**	1.20 (0.99,1.46)	**1.29 (1.07,1.55)**	0.96 (0.79,1.15)	1.05 (0.49,2.25)	**1.57 (1.35,1.82)**	**1.35 (1.13,1.61)**	**1.35 (1.17,1.55)**
**0.74 (0.58,0.95)**	**PPX**	**0.70 (0.50,0.98)**	0.93 (0.61,1.40)	1.04 (0.75,1.45)	0.89 (0.68,1.17)	0.96 (0.70,1.30)	**0.71 (0.52,0.96)**	0.78 (0.35,1.73)	1.16 (0.87,1.54)	1.00 (0.74,1.35)	1.00 (0.75,1.32)
1.06 (0.76,1.46)	**1.43 (1.03,1.98)**	**PPX ER**	1.32 (0.83,2.10)	**1.49 (1.00,2.21)**	1.27 (0.88,1.83)	1.36 (0.94,1.98)	1.01 (0.69,1.47)	1.11 (0.49,2.54)	**1.65 (1.15,2.37)**	1.43 (0.98,2.07)	1.42 (1.00,2.03)
0.80 (0.57,1.12)	1.08 (0.71,1.63)	0.76 (0.48,1.21)	**ROP**	1.13 (0.76,1.68)	0.96 (0.66,1.42)	1.03 (0.71,1.51)	0.76 (0.52,1.12)	0.84 (0.37,1.93)	1.25 (0.87,1.81)	1.08 (0.74,1.58)	1.08 (0.75,1.55)
**0.71 (0.57,0.88)**	0.96 (0.69,1.33)	**0.67 (0.45,1.00)**	0.89 (0.59,1.32)	**ROP ER**	0.85 (0.64,1.15)	0.92 (0.69,1.22)	**0.68 (0.51,0.90)**	0.75 (0.34,1.64)	1.11 (0.85,1.45)	0.96 (0.72,1.27)	0.96 (0.74,1.24)
0.83 (0.68,1.01)	1.12 (0.85,1.47)	0.79 (0.55,1.13)	1.04 (0.70,1.53)	1.17 (0.87,1.57)	**RTG patch**	1.07 (0.82,1.40)	0.79 (0.61,1.04)	0.87 (0.40,1.91)	**1.30 (1.02,1.66)**	1.12 (0.86,1.46)	1.12 (0.88,1.43)
**0.78 (0.65,0.93)**	1.05 (0.77,1.42)	0.73 (0.50,1.07)	0.97 (0.66,1.42)	1.09 (0.82,1.46)	0.93 (0.71,1.22)	**RAS**	**0.74 (0.57,0.96)**	0.81 (0.37,1.78)	1.21 (0.98,1.51)	1.05 (0.81,1.35)	1.05 (0.83,1.32)
1.05 (0.87,1.26)	**1.41 (1.04,1.92)**	0.99 (0.68,1.44)	1.31 (0.89,1.92)	**1.48 (1.11,1.97)**	1.26 (0.96,1.65)	**1.35 (1.04,1.75)**	**SAF**	1.10 (0.50,2.40)	**1.64 (1.29,2.08)**	**1.41 (1.09,1.83)**	**1.41 (1.12,1.78)**
0.95 (0.45,2.03)	1.28 (0.58,2.85)	0.90 (0.39,2.06)	1.19 (0.52,2.72)	1.34 (0.61,2.96)	1.15 (0.52,2.51)	1.23 (0.56,2.68)	0.91 (0.42,1.99)	**SEL**	1.49 (0.69,3.23)	1.28 (0.59,2.80)	1.28 (0.59,2.78)
**0.64 (0.55,0.74)**	0.86 (0.65,1.15)	**0.61 (0.42,0.87)**	0.80 (0.55,1.15)	0.90 (0.69,1.18)	**0.77 (0.60,0.98)**	0.82 (0.66,1.03)	**0.61 (0.48,0.77)**	0.67 (0.31,1.46)	**ENT**	0.86 (0.70,1.06)	0.86 (0.71,1.05)
**0.74 (0.62,0.88)**	1.00 (0.74,1.35)	0.70 (0.48,1.02)	0.93 (0.63,1.35)	1.04 (0.79,1.39)	0.89 (0.69,1.16)	0.96 (0.74,1.23)	**0.71 (0.55,0.92)**	0.78 (0.36,1.70)	1.16 (0.94,1.43)	**OPC**	1.00 (0.80,1.25)
**0.74 (0.64,0.85)**	1.00 (0.75,1.33)	0.70 (0.49,1.00)	0.93 (0.64,1.33)	1.04 (0.80,1.36)	0.89 (0.70,1.14)	0.96 (0.76,1.20)	**0.71 (0.56,0.89)**	0.78 (0.36,1.69)	1.16 (0.95,1.41)	1.00 (0.80,1.25)	**ISD**

Global inconsistency test: Chi-square=0.80, P=0.977 OR (95%CI)

3E) Dyskinesia

**PBO**	**2.75 (1.96,3.88)**	**2.38 (1.47,3.84)**	**2.72 (1.58,4.67)**	**5.77 (3.24,10.28)**	**2.42 (1.72,3.41)**	**1.80 (1.31,2.46)**	**2.16 (1.60,2.90)**	0.71 (0.23,2.23)	**2.15 (1.72,2.68)**	**3.43 (2.48,4.76)**	**1.98 (1.61,2.43)**
**0.36 (0.26,0.51)**	**PPX**	0.86 (0.55,1.36)	0.99 (0.52,1.87)	**2.09 (1.07,4.10)**	0.88 (0.59,1.31)	0.65 (0.41,1.04)	0.78 (0.50,1.23)	**0.26 (0.08,0.85)**	0.78 (0.52,1.17)	1.25 (0.78,2.00)	0.72 (0.48,1.07)
**0.42 (0.26,0.68)**	1.16 (0.73,1.83)	**PPX ER**	1.14 (0.55,2.36)	**2.43 (1.14,5.14)**	1.02 (0.59,1.77)	0.76 (0.43,1.34)	0.91 (0.52,1.59)	0.30 (0.09,1.03)	0.90 (0.53,1.53)	1.44 (0.81,2.58)	0.83 (0.49,1.40)
**0.37 (0.21,0.63)**	1.01 (0.53,1.92)	0.87 (0.42,1.80)	**ROP**	2.12 (0.96,4.69)	0.89 (0.47,1.69)	0.66 (0.35,1.24)	0.79 (0.43,1.47)	**0.26 (0.07,0.93)**	0.79 (0.44,1.42)	1.26 (0.67,2.38)	0.73 (0.41,1.30)
**0.17 (0.10,0.31)**	**0.48 (0.24,0.93)**	**0.41 (0.19,0.87)**	0.47 (0.21,1.04)	**ROP ER**	**0.42 (0.21,0.82)**	**0.31 (0.16,0.60)**	**0.37 (0.20,0.72)**	**0.12 (0.03,0.44)**	**0.37 (0.20,0.69)**	0.60 (0.31,1.16)	**0.34 (0.19,0.63)**
**0.41 (0.29,0.58)**	1.14 (0.76,1.70)	0.98 (0.57,1.71)	1.12 (0.59,2.13)	**2.38 (1.22,4.67)**	**RTG patch**	0.74 (0.47,1.18)	0.89 (0.57,1.40)	**0.30 (0.09,0.97)**	0.89 (0.59,1.34)	1.42 (0.88,2.28)	0.82 (0.55,1.22)
**0.56 (0.41,0.76)**	1.53 (0.96,2.44)	1.32 (0.74,2.35)	1.51 (0.81,2.83)	**3.21 (1.66,6.19)**	1.35 (0.84,2.14)	**RAS**	1.20 (0.78,1.85)	0.40 (0.12,1.29)	1.20 (0.83,1.72)	1.91 (1.22,2.99)	1.10 (0.76,1.60)
**0.46 (0.34,0.62)**	1.28 (0.81,2.01)	1.10 (0.63,1.94)	1.26 (0.68,2.34)	**2.68 (1.40,5.13)**	1.12 (0.71,1.77)	0.83 (0.54,1.29)	**SAF**	0.33 (0.10,1.07)	1.00 (0.69,1.45)	**1.59 (1.03,2.48)**	0.92 (0.64,1.32)
1.40 (0.45,4.36)	**3.85 (1.18,12.63)**	3.33 (0.97,11.42)	**3.80 (1.08,13.39)**	**8.07 (2.25,28.89)**	**3.38 (1.03,11.10)**	2.52 (0.77,8.18)	3.01 (0.93,9.76)	**SEL**	3.01 (0.94,9.58)	**4.81 (1.47,15.67)**	2.77 (0.87,8.78)
**0.47 (0.37,0.58)**	1.28 (0.85,1.93)	1.11 (0.65,1.88)	1.26 (0.70,2.27)	**2.68 (1.44,4.99)**	1.13 (0.75,1.69)	0.84 (0.58,1.20)	1.00 (0.69,1.45)	0.33 (0.10,1.06)	**ENT**	**1.60 (1.12,2.29)**	0.92 (0.64,1.32)
**0.29 (0.21,0.40)**	0.80 (0.50,1.29)	0.69 (0.39,1.24)	0.79 (0.42,1.49)	1.68 (0.87,3.26)	0.70 (0.44,1.13)	0.52 (0.33,0.82)	**0.63 (0.40,0.97)**	**0.21 (0.06,0.68)**	**0.63 (0.44,0.90)**	**OPC**	**0.58 (0.39,0.84)**
**0.51 (0.41,0.62)**	1.39 (0.93,2.08)	1.20 (0.71,2.03)	1.37 (0.77,2.45)	**2.92 (1.58,5.39)**	1.22 (0.82,1.83)	0.91 (0.63,1.32)	1.09 (0.76,1.56)	0.36 (0.11,1.15)	1.09 (0.82,1.45)	**1.74 (1.19,2.54)**	**ISD**

Global inconsistency test: Chi-square=1.12, P=0.953 OR (95%CI)

3F) Hallucination

**PBO**	**3.76 (1.94,7.29)**	**5.08 (1.47,17.52)**	1.71 (0.44,6.61)	2.96 (0.94,9.34)	**3.55 (1.56,8.07)**	1.41 (0.61,3.26)	1.30 (0.61,2.79)	2.87 (0.24,33.71)	0.98 (0.52,1.86)	**4.74 (1.96,11.46)**	1.84 (0.97,3.49)
**0.27 (0.14,0.51)**	**PPX**	1.35 (0.43,4.21)	0.45 (0.10,2.05)	0.79 (0.21,2.95)	0.94 (0.40,2.24)	0.37 (0.13,1.08)	**0.35 (0.13,0.95)**	0.76 (0.06,9.78)	**0.26 (0.10,0.66)**	1.26 (0.42,3.81)	0.49 (0.20,1.21)
**0.20 (0.06,0.68)**	0.74 (0.24,2.31)	**PPX ER**	0.34 (0.05,2.10)	0.58 (0.11,3.14)	0.70 (0.18,2.79)	0.28 (0.06,1.23)	0.26 (0.06,1.09)	0.57 (0.04,8.90)	**0.19 (0.05,0.79)**	0.93 (0.20,4.28)	0.36 (0.09,1.44)
0.58 (0.15,2.26)	2.20 (0.49,9.90)	2.97 (0.48,18.56)	**ROP**	1.73 (0.29,10.19)	2.08 (0.43,10.08)	0.82 (0.17,4.04)	0.76 (0.16,3.59)	1.68 (0.10,27.85)	0.57 (0.13,2.55)	2.77 (0.55,13.91)	1.07 (0.24,4.79)
0.34 (0.11,1.07)	1.27 (0.34,4.78)	1.72 (0.32,9.28)	0.58 (0.10,3.41)	**ROP ER**	1.20 (0.29,4.93)	0.48 (0.11,1.98)	0.44 (0.11,1.75)	0.97 (0.06,14.73)	0.33 (0.09,1.24)	1.60 (0.38,6.84)	0.62 (0.17,2.32)
**0.28 (0.12,0.64)**	1.06 (0.45,2.51)	1.43 (0.36,5.71)	0.48 (0.10,2.34)	0.83 (0.20,3.41)	**RTG patch**	0.40 (0.12,1.28)	0.37 (0.12,1.12)	0.81 (0.06,10.85)	**0.28 (0.10,0.79)**	1.33 (0.40,4.47)	0.52 (0.18,1.45)
0.71 (0.31,1.64)	2.67 (0.92,7.73)	3.61 (0.81,15.98)	1.21 (0.25,5.96)	2.10 (0.51,8.70)	2.52 (0.78,8.12)	**RAS**	0.93 (0.30,2.87)	2.04 (0.15,27.50)	0.70 (0.27,1.78)	**3.36 (1.02,11.07)**	1.31 (0.46,3.73)
0.77 (0.36,1.64)	**2.89 (1.06,7.88)**	3.90 (0.92,16.59)	1.31 (0.28,6.18)	2.27 (0.57,8.99)	2.72 (0.89,8.31)	1.08 (0.35,3.35)	**SAF**	2.20 (0.17,28.99)	0.75 (0.28,2.04)	**3.63 (1.13,11.65)**	1.41 (0.52,3.80)
0.35 (0.03,4.09)	1.31 (0.10,16.77)	1.77 (0.11,27.85)	0.60 (0.04,9.88)	1.03 (0.07,15.59)	1.24 (0.09,16.58)	0.49 (0.04,6.62)	0.45 (0.03,5.97)	**SEL**	0.34 (0.03,4.34)	1.65 (0.12,22.57)	0.64 (0.05,8.16)
1.02 (0.54,1.93)	**3.84 (1.50,9.80)**	**5.19 (1.26,21.28)**	1.75 (0.39,7.78)	3.02 (0.80,11.30)	**3.62 (1.26,10.40)**	1.44 (0.56,3.69)	1.33 (0.49,3.60)	2.93 (0.23,37.31)	**ENT**	**4.83 (1.81,12.88)**	1.88 (0.75,4.71)
**0.21 (0.09,0.51)**	0.79 (0.26,2.40)	1.07 (0.23,4.93)	0.36 (0.07,1.81)	0.62 (0.15,2.66)	0.75 (0.22,2.51)	**0.30 (0.09,0.98)**	**0.28 (0.09,0.88)**	0.61 (0.04,8.30)	**0.21 (0.08,0.55)**	**OPC**	0.39 (0.13,1.16)
0.54 (0.29,1.03)	2.05 (0.82,5.08)	2.76 (0.69,11.00)	0.93 (0.21,4.15)	1.61 (0.43,5.98)	1.93 (0.69,5.42)	0.77 (0.27,2.19)	0.71 (0.26,1.91)	1.56 (0.12,19.89)	0.53 (0.21,1.34)	2.58 (0.86,7.69)	**ISD**

Global inconsistency test: Chi-square=3.69, P=0.450 OR (95%CI)

3G) Orthostatic hypotension

**PBO**	0.95 (0.59,1.52)	1.58 (0.43,5.78)	1.10 (0.49,2.48)	1.55 (0.67,3.59)	**0.40 (0.24,0.68)**	**3.93 (1.58,9.75)**	0.91 (0.44,1.88)	1.00 (0.09,11.30)	2.11 (0.93,4.75)	1.93 (0.54,6.91)	1.52 (0.67,3.47)
1.05 (0.66,1.69)	**PPX**	1.67 (0.46,6.10)	1.16 (0.45,2.97)	1.63 (0.62,4.28)	**0.42 (0.23,0.78)**	**4.14 (1.49,11.53)**	0.95 (0.40,2.28)	1.05 (0.09,12.47)	2.22 (0.87,5.69)	2.03 (0.52,7.92)	1.60 (0.62,4.14)
0.63 (0.17,2.31)	0.60 (0.16,2.19)	**PPX ER**	0.70 (0.15,3.21)	0.98 (0.21,4.58)	**0.25 (0.06,1.00)**	2.48 (0.51,12.08)	0.57 (0.13,2.53)	0.63 (0.04,9.88)	1.33 (0.29,6.14)	1.22 (0.20,7.50)	0.96 (0.21,4.46)
0.91 (0.40,2.05)	0.86 (0.34,2.21)	1.44 (0.31,6.65)	**ROP**	1.41 (0.44,4.54)	**0.36 (0.14,0.96)**	**3.57 (1.05,12.09)**	0.82 (0.28,2.46)	0.91 (0.07,11.73)	1.91 (0.60,6.05)	1.75 (0.39,7.96)	1.38 (0.43,4.40)
0.65 (0.28,1.50)	0.61 (0.23,1.61)	1.02 (0.22,4.80)	0.71 (0.22,2.30)	**ROP ER**	**0.26 (0.10,0.70)**	2.54 (0.73,8.77)	0.59 (0.19,1.79)	0.65 (0.05,8.42)	1.36 (0.42,4.39)	1.25 (0.27,5.75)	0.98 (0.30,3.19)
**2.51 (1.48,4.25)**	**2.38 (1.28,4.41)**	**3.97 (1.00,15.74)**	**2.76 (1.05,7.28)**	**3.88 (1.44,10.48)**	**RTG patch**	**9.85 (3.44,28.16)**	2.27 (0.92,5.59)	2.51 (0.21,29.99)	**5.28 (2.00,13.92)**	**4.83 (1.22,19.22)**	**3.81 (1.43,10.14)**
**0.25 (0.10,0.63)**	**0.24 (0.09,0.67)**	0.40 (0.08,1.96)	**0.28 (0.08,0.95)**	0.39 (0.11,1.36)	**0.10 (0.04,0.29)**	**RAS**	**0.23 (0.07,0.74)**	0.25 (0.02,3.39)	0.54 (0.20,1.42)	0.49 (0.11,2.18)	0.39 (0.11,1.32)
1.10 (0.53,2.29)	1.05 (0.44,2.50)	1.75 (0.40,7.74)	1.22 (0.41,3.63)	1.71 (0.56,5.21)	0.44 (0.18,1.08)	**4.34 (1.35,13.92)**	**SAF**	1.10 (0.09,13.90)	2.33 (0.78,6.94)	2.13 (0.49,9.26)	1.68 (0.56,5.05)
1.00 (0.09,11.30)	0.95 (0.08,11.21)	1.58 (0.10,24.75)	1.10 (0.09,14.20)	1.55 (0.12,20.15)	0.40 (0.03,4.77)	3.93 (0.29,52.32)	0.91 (0.07,11.39)	**SEL**	2.11 (0.16,27.18)	1.93 (0.12,29.85)	1.52 (0.12,19.68)
0.47 (0.21,1.07)	0.45 (0.18,1.15)	0.75 (0.16,3.47)	0.52 (0.17,1.65)	0.73 (0.23,2.37)	**0.19 (0.07,0.50)**	1.86 (0.70,4.94)	0.43 (0.14,1.28)	0.47 (0.04,6.13)	**ENT**	0.92 (0.25,3.41)	0.72 (0.23,2.30)
0.52 (0.14,1.86)	0.49 (0.13,1.92)	0.82 (0.13,5.06)	0.57 (0.13,2.59)	0.80 (0.17,3.70)	**0.21 (0.05,0.82)**	2.04 (0.46,9.06)	0.47 (0.11,2.04)	0.52 (0.03,8.04)	1.09 (0.29,4.07)	**OPC**	0.79 (0.17,3.60)
0.66 (0.29,1.50)	0.62 (0.24,1.61)	1.04 (0.22,4.84)	0.72 (0.23,2.31)	1.02 (0.31,3.31)	**0.26 (0.10,0.70)**	2.58 (0.76,8.82)	0.60 (0.20,1.79)	0.66 (0.05,8.52)	1.39 (0.43,4.41)	1.27 (0.28,5.79)	**ISD**

Global inconsistency test: Chi-square=4.62, P=0.329

OR (95%CI)

Supplementary Figure 3. League tables of the NMA results

A) The numbers are SMDs (95%CIs) for the change in daily off-time in the column-defining treatment compared with the row-defining treatment. B-G) The numbers are ORs (95%CIs) of each outcome in the column-defining treatment compared with the row-defining treatment. All numbers are shown after rounding off to two decimal places. 0.00 and -0.00 mean positive and negative values, respectively. The bold font indicates significant results. Results for global inconsistency test are also shown below each table, and no inconsistency was detected in any NMA.

Abbreviations: NMA, network meta-analysis; PBO, placebo; PPX, pramipexole; PPX ER, pramipexole extended release; ROP, ropinirole; ROP ER, ropinirole extended release; ROP patch, ropinirole transdermal patch; RTG patch, rotigotine transdermal patch; RAS, rasagiline; SAF, safinamide; SEL, selegiline; ENT, entacapone; OPC, opicapone; ISD, istradefylline; SMD, standardized mean difference; CI, confidence interval; OR, odds ratio; AE, adverse event.

4A) Change in daily off-time

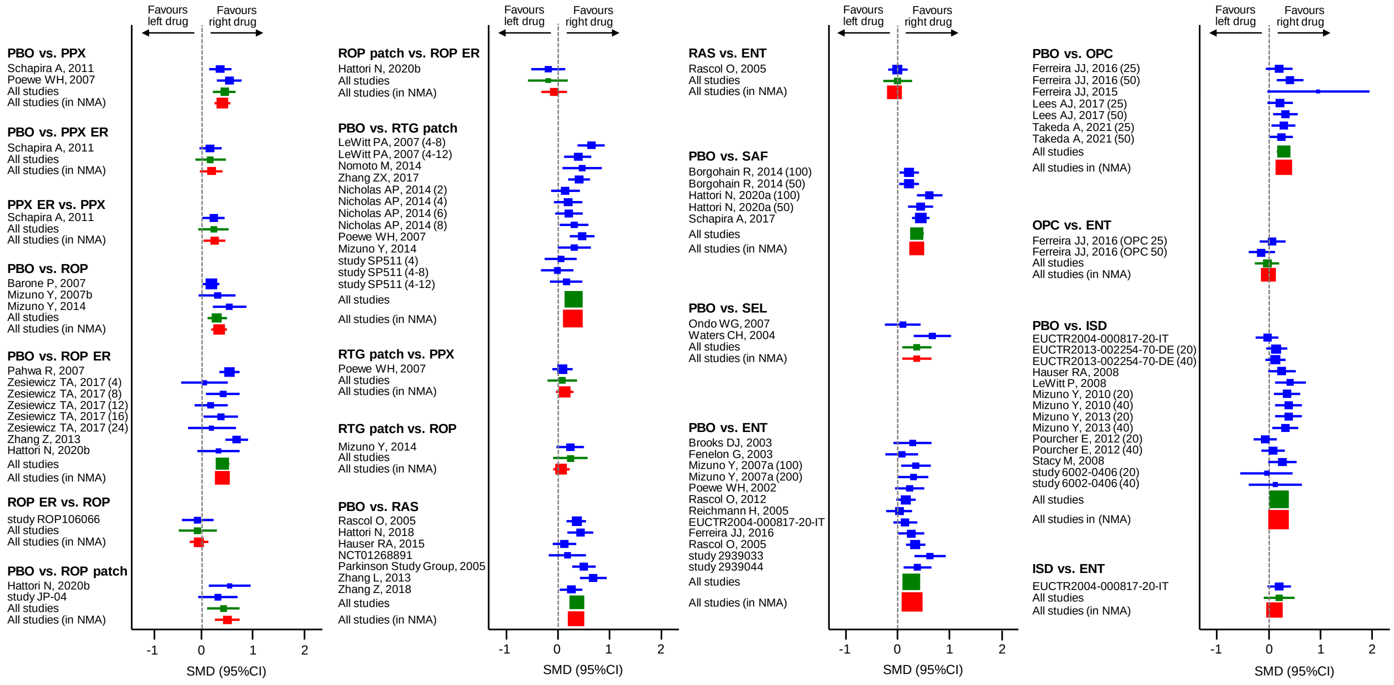

4B) Discontinuation due to all causes

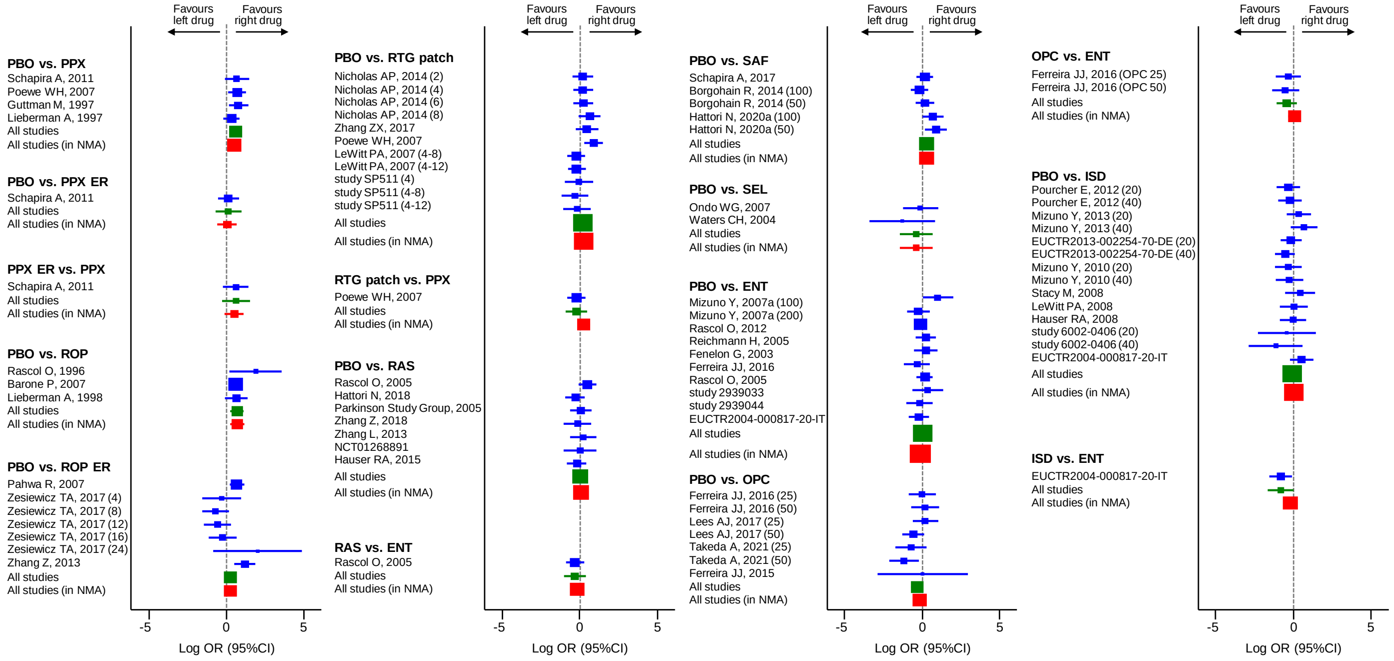

4C) Discontinuation due to AEs

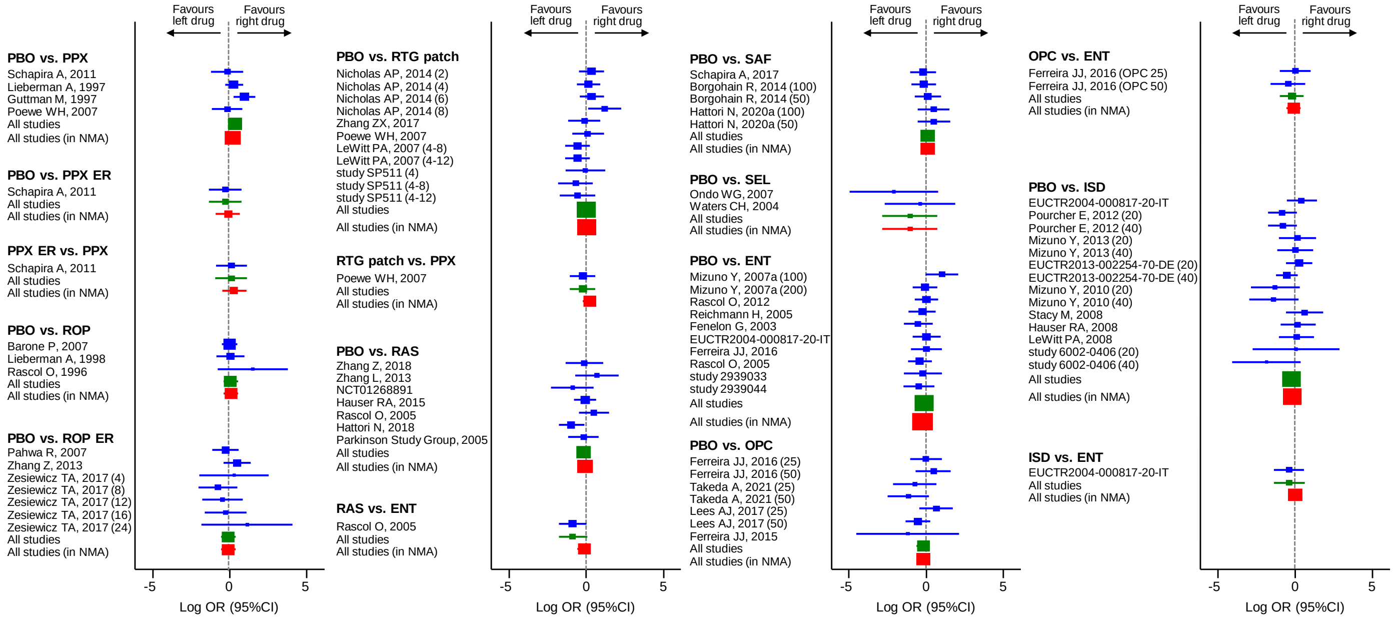

4D) AEs

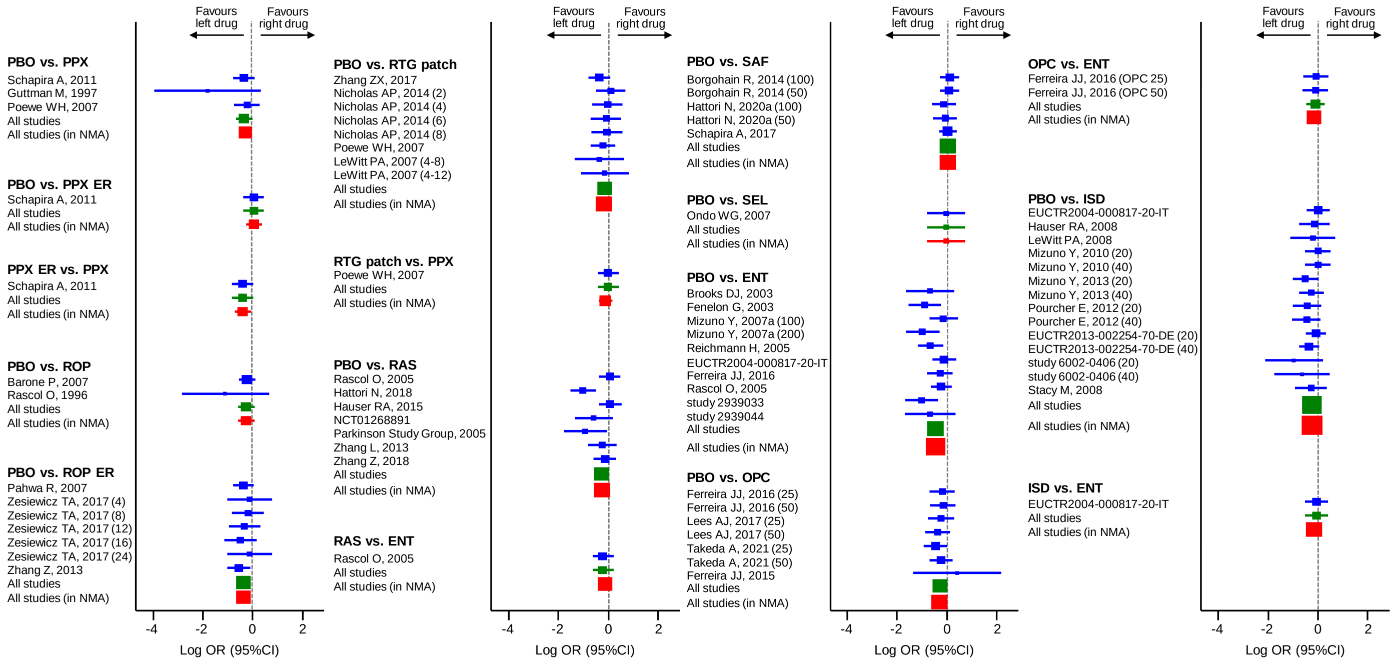

4E) Dyskinesia

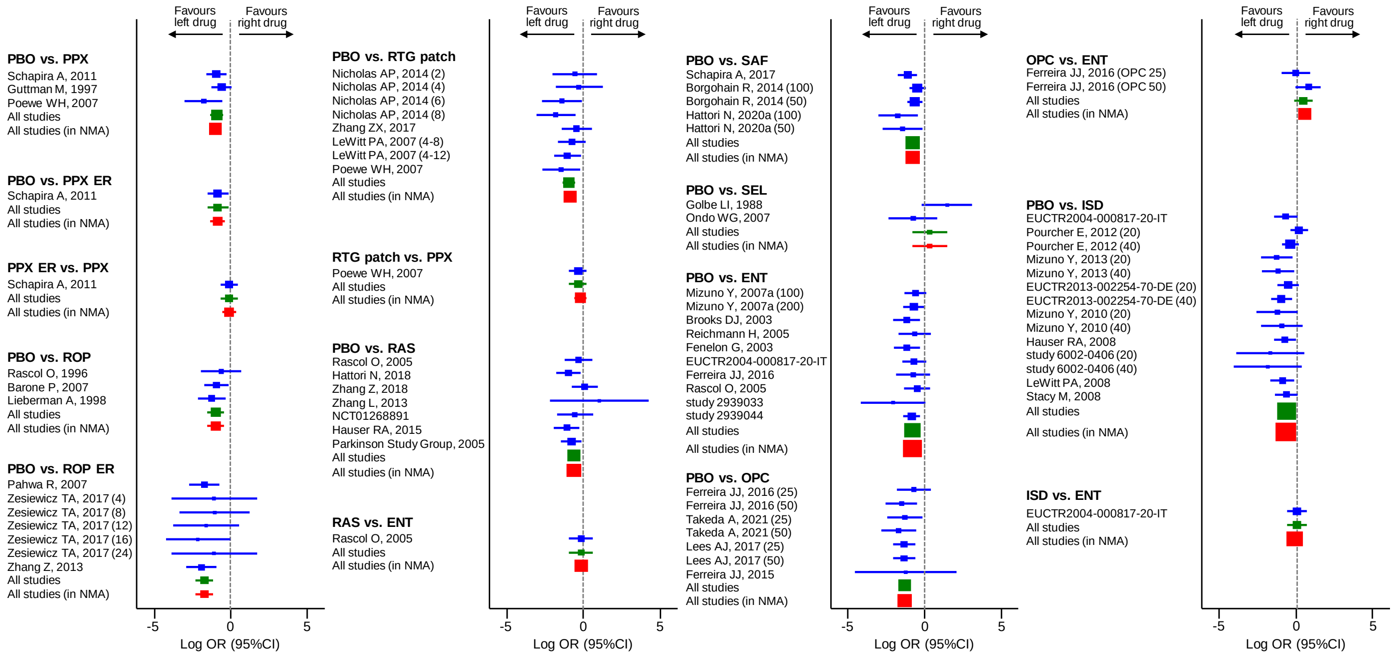

4F) Hallucination

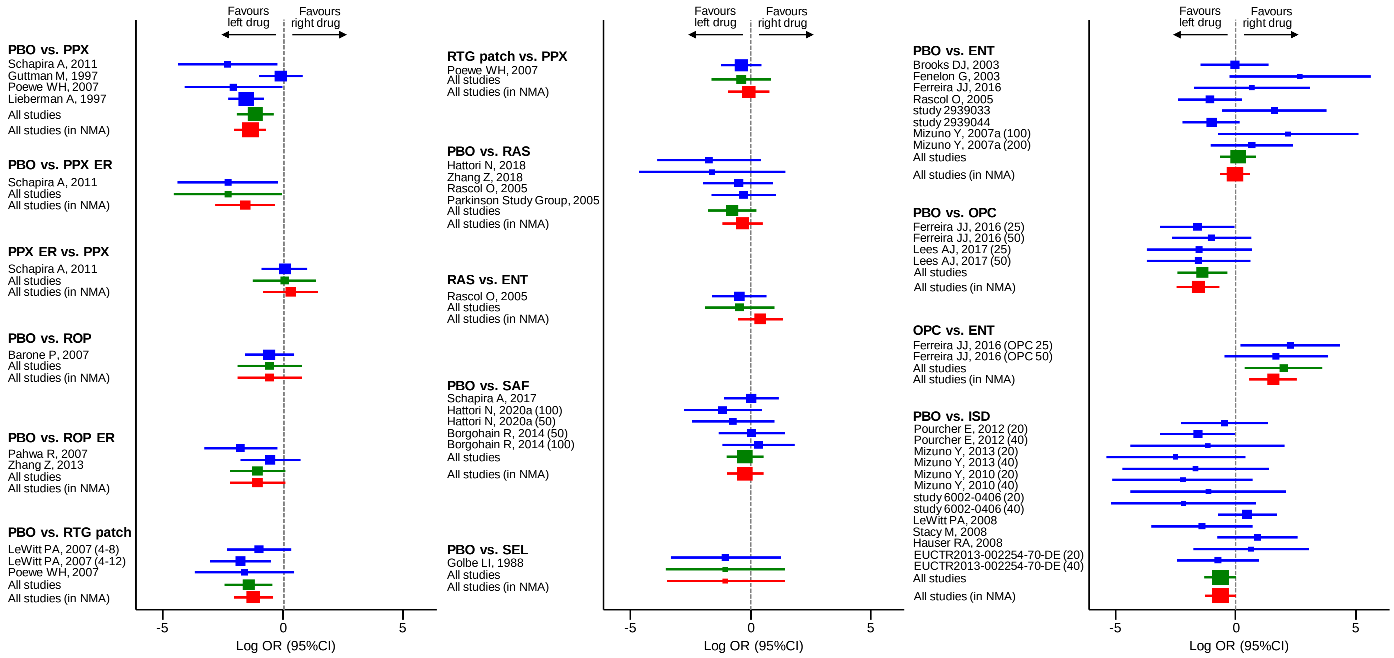

4G) Orthostatic hypotension

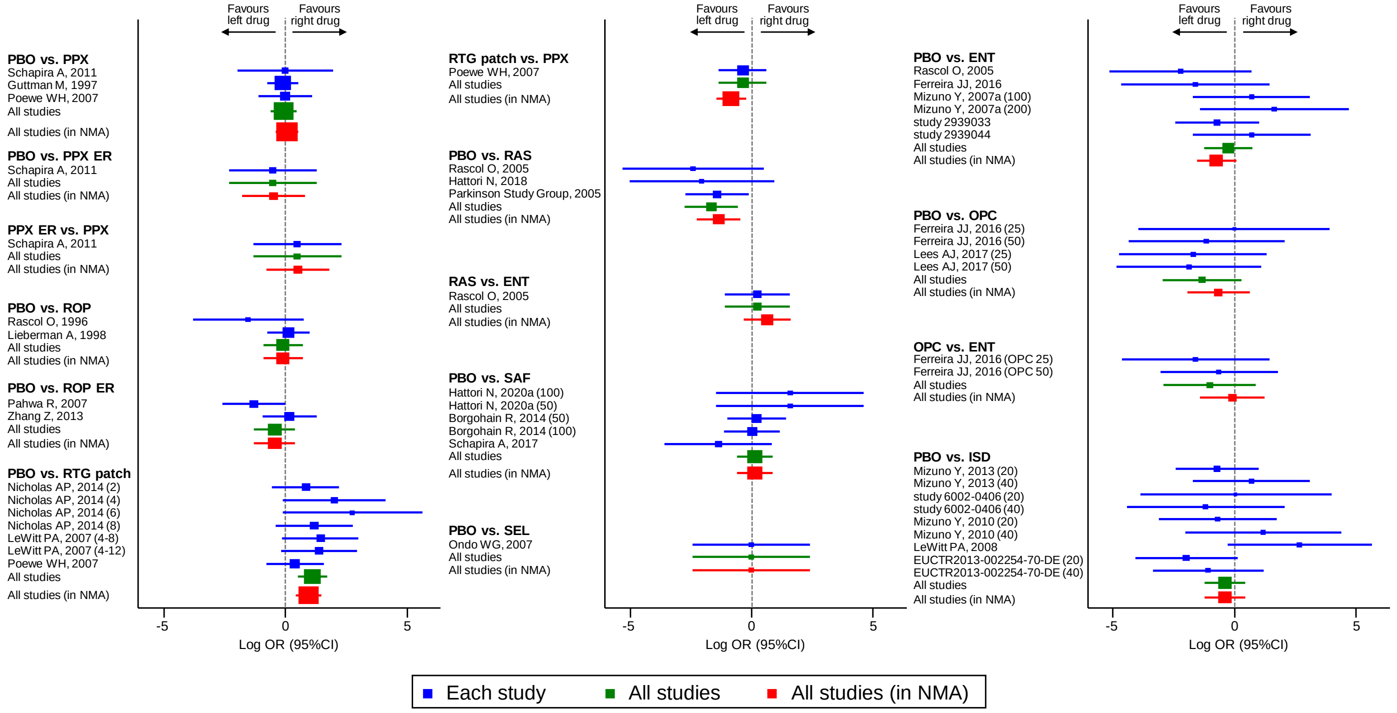

Supplementary Figure 4. Forest plots of the NMA results

Forest plots were generated to illustrate the effect of each study, the pooled effect of all studies in the direct comparison, and the pooled effect of all studies in NMA along with 95% CI. The boxes indicate estimated effect sizes and the size of the boxes corresponds to the number of studies. The horizontal lines indicates 95% CI. Numbers within parenthesis by each study ID indicates the dosage of study drugs, and detail information of each study are available in eTable 2.

Abbreviations: NMA, network meta-analysis; PBO, placebo; PPX, pramipexole; PPX ER, pramipexole extended release; ROP, ropinirole; ROP ER, ropinirole extended release; ROP patch, ropinirole transdermal patch; RTG patch, rotigotine transdermal patch; RAS, rasagiline; SAF, safinamide; SEL, selegiline; ENT, entacapone; OPC, opicapone; ISD, istradefylline; SMD, standardized mean difference; CI, confidence interval; OR, odds ratio; AE, adverse event.

Supplementary Table 2. Heterogeneity of each comparison

A) Change in daily off-time

	Heterogeneity with random effect model			
	**Chi-squared**	**d.f.**	**P-value**	**I-squared (%)**
Pramipexole vs Placebo	1.12	1	0.291	10.3
Pramipexole ER vs Placebo	-			
Pramipexole ER vs Pramipexole	-			
Ropinirole vs Placebo	3.77	2	0.152	47.0
Ropinirole ER vs Placebo	12.19	7	0.095	42.6
Ropinirole ER vs Ropinirole	-			
Ropinirole patch vs Placebo	0.62	1	0.433	0.0
Ropinirole patch vs Ropinirole ER	-			
Rotigotine patch vs Placebo	20.12	12	0.065	40.4
Rotigotine patch vs Pramipexole	-			
Rotigotine patch vs Ropinirole	-			
Rasagiline vs Placebo	13.76	6	0.032	56.4
Rasagiline vs Entacapone	-			
Safinamide vs Placebo	9.57	4	0.048	58.2
Selegiline vs Placebo	5.18	1	0.023	80.7
Entacapone vs Placebo	13.84	11	0.242	20.5
Opicapone vs Placebo	3.85	6	0.697	0.0
Opicapone vs Entacapone	1.31	1	0.252	23.9
Istradefylline vs Placebo	21.03	13	0.072	38.2
Istradefylline vs Entacapone	-			

B) Discontinuation due to all causes

	Heterogeneity with random effect model			
	**Chi-squared**	**d.f.**	**P-value**	**I-squared (%)**
Pramipexole vs Placebo	0.85	3	0.838	0.0
Pramipexole ER vs Placebo	-			
Pramipexole ER vs Pramipexole	-			
Ropinirole vs Placebo	1.61	2	0.448	0.0
Ropinirole ER vs Placebo	15.29	6	0.018	60.8
Rotigotine patch vs Placebo	9.42	10	0.493	0.0
Rotigotine patch vs Pramipexole	-			
Rasagiline vs Placebo	3.55	6	0.737	0.0
Rasagiline vs Entacapone	-			
Safinamide vs Placebo	4.79	4	0.309	16.5
Selegiline vs Placebo	0.90	1	0.342	0.0
Entacapone vs Placebo	6.13	9	0.727	0.0
Opicapone vs Placebo	6.31	6	0.389	4.9
Opicapone vs Entacapone	0.08	1	0.781	0.0
Istradefylline vs Placebo	10.10	13	0.685	0.0
Istradefylline vs Entacapone	-			

C) Discontinuation due to AEs

	Heterogeneity with random effect model			
	**Chi-squared**	**d.f.**	**P-value**	**I-squared (%)**
Pramipexole vs Placebo	2.45	3	0.484	0.0
Pramipexole ER vs Placebo	-			
Pramipexole ER vs Pramipexole	-			
Ropinirole vs Placebo	1.30	2	0.522	0.0
Ropinirole ER vs Placebo	3.50	6	0.744	0.0
Rotigotine patch vs Placebo	10.32	10	0.413	3.1
Rotigotine patch vs Pramipexole	-			
Rasagiline vs Placebo	6.68	6	0.351	10.2
Rasagiline vs Entacapone	-			
Safinamide vs Placebo	1.64	4	0.802	0.0
Selegiline vs Placebo	0.80	1	0.371	0.0
Entacapone vs Placebo	5.24	9	0.796	0.0
Opicapone vs Placebo	6.36	6	0.384	5.6
Opicapone vs Entacapone	0.31	1	0.575	0.0
Istradefylline vs Placebo	13.93	13	0.379	6.7
Istradefylline vs Entacapone	-			

D) AEs

	Heterogeneity with random effect model			
	**Chi-squared**	**d.f.**	**P-value**	**I-squared (%)**
Pramipexole vs Placebo	0.14	2	0.934	0.0
Pramipexole ER vs Placebo	-			
Pramipexole ER vs Pramipexole	-			
Ropinirole vs Placebo	0.04	1	0.845	0.0
Ropinirole ER vs Placebo	0.22	6	1.000	0.0
Rotigotine patch vs Placebo	0.74	7	0.998	0.0
Rotigotine patch vs Pramipexole	-			
Rasagiline vs Placebo	3.61	6	0.729	0.0
Rasagiline vs Entacapone	-			
Safinamide vs Placebo	0.17	4	0.997	0.0
Selegiline vs Placebo	-			
Entacapone vs Placebo	3.15	9	0.958	0.0
Opicapone vs Placebo	0.50	6	0.998	0.0
Opicapone vs Entacapone	0.00	1	0.966	0.0
Istradefylline vs Placebo	1.66	13	1.000	0.0
Istradefylline vs Entacapone	-			

E) Dyskinesia

	Heterogeneity with random effect model			
	**Chi-squared**	**d.f.**	**P-value**	**I-squared (%)**
Pramipexole vs Placebo	4.42	2	0.11	54.7
Pramipexole ER vs Placebo	-			
Pramipexole ER vs Pramipexole	-			
Ropinirole vs Placebo	0.66	2	0.719	0.0
Ropinirole ER vs Placebo	0.67	6	0.995	0.0
Rotigotine patch vs Placebo	4.14	7	0.763	0.0
Rotigotine patch vs Pramipexole	-			
Rasagiline vs Placebo	4.97	6	0.547	0.0
Rasagiline vs Entacapone	-			
Safinamide vs Placebo	6.51	4	0.164	38.5
Selegiline vs Placebo	2.97	1	0.085	66.3
Entacapone vs Placebo	3.77	9	0.926	0.0
Opicapone vs Placebo	1.56	6	0.955	0.0
Opicapone vs Entacapone	1.28	1	0.259	21.6
Istradefylline vs Placebo	14.14	13	0.364	8.0
Istradefylline vs Entacapone	-			

F) Hallucination

	Heterogeneity with random effect model			
	Chi-squared	d.f.	P-value	I-squared (%)
Pramipexole vs Placebo	8.09	3	0.044	62.9
Pramipexole ER vs Placebo	-			
Pramipexole ER vs Pramipexole	-			
Ropinirole vs Placebo	-			
Ropinirole ER vs Placebo	1.48	1	0.223	32.5
Rotigotine patch vs Placebo	0.54	2	0.763	0.0
Rotigotine patch vs Pramipexole	-			
Rasagiline vs Placebo	1.62	3	0.656	0.0
Rasagiline vs Entacapone	-			
Safinamide vs Placebo	2.35	4	0.671	0.0
Selegiline vs Placebo	-			
Entacapone vs Placebo	12.74	7	0.079	45.1
Opicapone vs Placebo	0.28	3	0.963	0.0
Opicapone vs Entacapone	0.12	1	0.727	0.0
Istradefylline vs Placebo	13.39	12	0.341	10.4

G) Orthostatic hypotension

	Heterogeneity with random effect model			
	Chi-squared	d.f.	P-value	I-squared (%)
Pramipexole vs Placebo	0.01	2	0.997	0.0
Pramipexole ER vs Placebo	-			
Pramipexole ER vs Pramipexole	-			
Ropinirole vs Placebo	1.42	1	0.234	29.4
Ropinirole ER vs Placebo	2.71	1	0.100	63.1
Rotigotine patch vs Placebo	3.80	6	0.704	0.0
Rotigotine patch vs Pramipexole	-			
Rasagiline vs Placebo	0.56	2	0.757	0.0
Rasagiline vs Entacapone	-			
Safinamide vs Placebo	3.53	4	0.473	0.0
Selegiline vs Placebo	-			
Entacapone vs Placebo	5.37	5	0.372	6.9
Opicapone vs Placebo	0.11	2	0.949	0.0
Opicapone vs Entacapone	0.23	1	0.629	0.0
Istradefylline vs Placebo	8.37	7	0.301	16.4

Using the data from the direct comparisons, the heterogeneity test and I-squared values were calculated for each drug comparison

Abbreviation: d.f., degrees of freedom; ER, extended release; ropinirole patch, ropinirole transdermal patch; rotigotine patch, rotigotine transdermal patch; AE, adverse event.

Supplementary Table 3. Inconsistency test result for direct and indirect comparison

A) Change in daily off-time

Side		Direct		Indirect		Difference		
		Coef.	Std. Err.	Coef.	Std. Err.	Coef.	Std. Err.	P>z
Placebo	Pramipexole	0.451	0.107	0.355	0.120	0.096	0.161	0.550
Placebo	Pramipexole ER	0.161	0.147	0.213	0.171	-0.053	0.226	0.816
Pramipexole ER	Pramipexole	0.203	0.148	0.256	0.171	-0.053	0.226	0.816
Placebo	Ropinirole	0.304	0.093	0.450	0.129	-0.146	0.159	0.359
Placebo	Ropinirole ER	0.409	0.067	0.334	0.158	0.075	0.172	0.662
Ropinirole ER	Ropinirole	-0.067	0.188	-0.036	0.106	-0.031	0.216	0.886
Placebo	Ropinirole patch	0.404	0.158	0.618	0.204	-0.213	0.258	0.408
Ropinirole patch	Ropinirole ER	-0.207	0.194	0.006	0.170	-0.213	0.258	0.408
Placebo	Rotigotine patch	0.298	0.048	0.234	0.125	0.064	0.134	0.633
Rotigotine patch	Pramipexole	0.066	0.140	0.149	0.106	-0.083	0.176	0.639
Rotigotine patch	Ropinirole	0.236	0.164	0.004	0.096	0.232	0.190	0.221
Placebo	Rasagiline	0.359	0.059	0.275	0.143	0.084	0.155	0.588
Rasagiline	Entacapone	0.009	0.137	-0.075	0.073	0.084	0.155	0.588
Placebo	Entacapone	0.277	0.048	0.332	0.083	-0.056	0.096	0.561
Placebo	Opicapone	0.291	0.064	0.326	0.123	-0.034	0.139	0.805
Opicapone	Entacapone	-0.032	0.115	0.002	0.077	-0.034	0.139	0.805
Placebo	Istradefylline	0.191	0.044	0.077	0.157	0.114	0.163	0.482
Istradefylline	Entacapone	0.206	0.151	0.091	0.062	0.114	0.163	0.482

B) Discontinuation due to all causes

Side		Direct		Indirect		Difference		
		Coef.	Std. Err.	Coef.	Std. Err.	Coef.	Std. Err.	P>z
Placebo	Pramipexole	0.600	0.194	0.146	0.328	0.454	0.381	0.234
Placebo	Pramipexole ER	0.100	0.427	-0.110	0.510	0.210	0.665	0.752
Pramipexole ER	Pramipexole	0.579	0.479	0.369	0.462	0.210	0.665	0.752
Placebo	Rotigotine patch	0.174	0.124	0.829	0.403	-0.655	0.422	0.121
Rotigotine patch	Pramipexole	-0.222	0.359	0.433	0.222	-0.655	0.422	0.121
Placebo	Rasagiline	0.022	0.165	0.223	0.395	-0.202	0.428	0.638
Rasagiline	Entacapone	-0.320	0.376	-0.118	0.205	-0.202	0.428	0.638
Placebo	Entacapone	0.032	0.129	-0.603	0.236	0.635	0.269	**0.018**
Placebo	Opicapone	-0.351	0.200	0.352	0.362	-0.703	0.413	0.089
Opicapone	Entacapone	-0.406	0.342	0.297	0.233	-0.703	0.413	0.089
Placebo	Istradefylline	-0.025	0.130	0.707	0.450	-0.733	0.468	0.118
Istradefylline	Entacapone	-0.773	0.434	-0.041	0.175	-0.733	0.468	0.118

C) Discontinuation due to AEs

Side		Direct		Indirect		Difference		
		Coef.	Std. Err.	Coef.	Std. Err.	Coef.	Std. Err.	P>z
Placebo	Pramipexole	0.348	0.206	-0.261	0.392	0.609	0.443	0.169
Placebo	Pramipexole ER	-0.225	0.552	0.166	0.570	-0.392	0.794	0.622
Pramipexole ER	Pramipexole	0.068	0.536	0.460	0.586	-0.392	0.794	0.622
Placebo	Rotigotine patch	-0.027	0.148	0.585	0.481	-0.611	0.503	0.224
Rotigotine patch	Pramipexole	-0.273	0.437	0.339	0.250	-0.611	0.503	0.224
Placebo	Rasagiline	-0.207	0.195	0.715	0.497	-0.922	0.534	0.085
Rasagiline	Entacapone	-0.886	0.478	0.035	0.240	-0.922	0.534	0.085
Placebo	Entacapone	-0.108	0.152	-0.691	0.290	0.583	0.327	0.074
Placebo	Opicapone	-0.211	0.233	-0.041	0.429	-0.170	0.489	0.729
Opicapone	Entacapone	-0.178	0.404	-0.008	0.274	-0.170	0.489	0.729
Placebo	Istradefylline	-0.256	0.158	0.213	0.548	-0.469	0.571	0.411
Istradefylline	Entacapone	-0.418	0.530	0.051	0.212	-0.469	0.571	0.411

D) AEs

Side		Direct		Indirect		Difference		
		Coef.	Std. Err.	Coef.	Std. Err.	Coef.	Std. Err.	P>z
Placebo	Pramipexole	-0.338	0.165	-0.249	0.191	-0.089	0.252	0.724
Placebo	Pramipexole ER	0.030	0.218	0.089	0.261	-0.059	0.340	0.863
Pramipexole ER	Pramipexole	-0.380	0.222	-0.321	0.257	-0.059	0.340	0.863
Placebo	Rotigotine patch	-0.164	0.108	-0.316	0.260	0.152	0.282	0.590
Rotigotine patch	Pramipexole	-0.025	0.216	-0.177	0.181	0.152	0.282	0.590
Placebo	Rasagiline	-0.274	0.102	-0.153	0.232	-0.121	0.253	0.633
Rasagiline	Entacapone	-0.282	0.218	-0.161	0.130	-0.121	0.253	0.633
Placebo	Entacapone	-0.460	0.093	-0.423	0.134	-0.037	0.163	0.820
Placebo	Opicapone	-0.283	0.102	-0.366	0.202	0.083	0.226	0.713
Opicapone	Entacapone	-0.093	0.184	-0.176	0.131	0.083	0.226	0.713
Placebo	Istradefylline	-0.291	0.075	-0.404	0.251	0.113	0.262	0.665
Istradefylline	Entacapone	-0.054	0.238	-0.168	0.110	0.114	0.262	0.665

E) Dyskinesia

Side		Direct		Indirect		Difference		
		Coef.	Std. Err.	Coef.	Std. Err.	Coef.	Std. Err.	P>z
Placebo	Pramipexole	-0.918	0.223	-1.162	0.279	0.244	0.358	0.495
Placebo	Pramipexole ER	-0.837	0.349	-0.895	0.344	0.058	0.490	0.906
Pramipexole ER	Pramipexole	-0.127	0.287	-0.185	0.397	0.058	0.490	0.906
Placebo	Rotigotine patch	-0.971	0.202	-0.614	0.354	-0.357	0.408	0.381
Rotigotine patch	Pramipexole	-0.313	0.292	0.044	0.284	-0.357	0.408	0.381
Placebo	Rasagiline	-0.588	0.173	-0.583	0.422	-0.005	0.456	0.992
Rasagiline	Entacapone	-0.182	0.405	-0.177	0.209	-0.005	0.456	0.992
Placebo	Entacapone	-0.772	0.132	-0.746	0.220	-0.027	0.257	0.917
Placebo	Opicapone	-1.268	0.191	-1.129	0.337	-0.138	0.387	0.721
Opicapone	Entacapone	0.377	0.315	0.515	0.225	-0.138	0.387	0.721
Placebo	Istradefylline	-0.666	0.111	-0.850	0.353	0.185	0.370	0.617
Istradefylline	Entacapone	0.066	0.332	-0.119	0.163	0.185	0.370	0.617

F) Hallucination

Side		Direct		Indirect		Difference		
		Coef.	Std. Err.	Coef.	Std. Err.	Coef.	Std. Err.	P>z
Placebo	Pramipexole	-1.125	0.379	-1.962	0.678	0.837	0.776	0.280
Placebo	Pramipexole ER	-2.330	1.153	-1.332	0.750	-0.998	1.376	0.468
Pramipexole ER	Pramipexole	0.069	0.664	1.067	1.205	-0.998	1.376	0.468
Placebo	Rotigotine patch	-1.481	0.512	-0.844	0.729	-0.637	0.891	0.475
Rotigotine patch	Pramipexole	-0.373	0.625	0.263	0.635	-0.637	0.891	0.475
Placebo	Rasagiline	-0.753	0.506	0.706	0.811	-1.459	0.959	0.128
Rasagiline	Entacapone	-0.501	0.733	0.957	0.619	-1.459	0.959	0.128
Placebo	Entacapone	0.156	0.374	-0.401	0.667	0.557	0.759	0.463
Placebo	Opicapone	-1.384	0.521	-2.059	0.897	0.675	1.037	0.515
Opicapone	Entacapone	2.008	0.828	1.333	0.624	0.675	1.037	0.515

G) Orthostatic hypotension

Side		Direct		Indirect		Difference		
		Coef.	Std. Err.	Coef.	Std. Err.	Coef.	Std. Err.	P>z
Placebo	Pramipexole	-0.081	0.269	0.585	0.536	-0.665	0.600	0.267
Placebo	Pramipexole ER	-0.495	0.919	-0.422	0.951	-0.073	1.323	0.956
Pramipexole ER	Pramipexole	0.477	0.919	0.550	0.951	-0.073	1.323	0.956
Placebo	Rotigotine patch	1.095	0.305	0.310	0.568	0.786	0.645	0.223
Rotigotine patch	Pramipexole	-0.388	0.503	-1.173	0.403	0.786	0.645	0.223
Placebo	Rasagiline	-1.651	0.561	-0.754	0.825	-0.897	0.998	0.369
Rasagiline	Entacapone	0.210	0.677	1.107	0.733	-0.897	0.998	0.369
Placebo	Entacapone	-0.257	0.507	-1.733	0.723	1.476	0.883	0.095
Placebo	Opicapone	-1.330	0.825	0.459	1.061	-1.789	1.344	0.183
Opicapone	Entacapone	-1.012	0.965	0.777	0.934	-1.789	1.344	0.183

Inconsistency between direct and indirect evidence was assessed using side-splitting approach. The red and bold font indicates significant results.

Abbreviations: Coef., coefficient; Std. Err., standard error; ER, extended release; ropinirole patch, ropinirole transdermal patch; rotigotine patch, rotigotine transdermal patch; AE, adverse event.

5A) Change in daily off-time

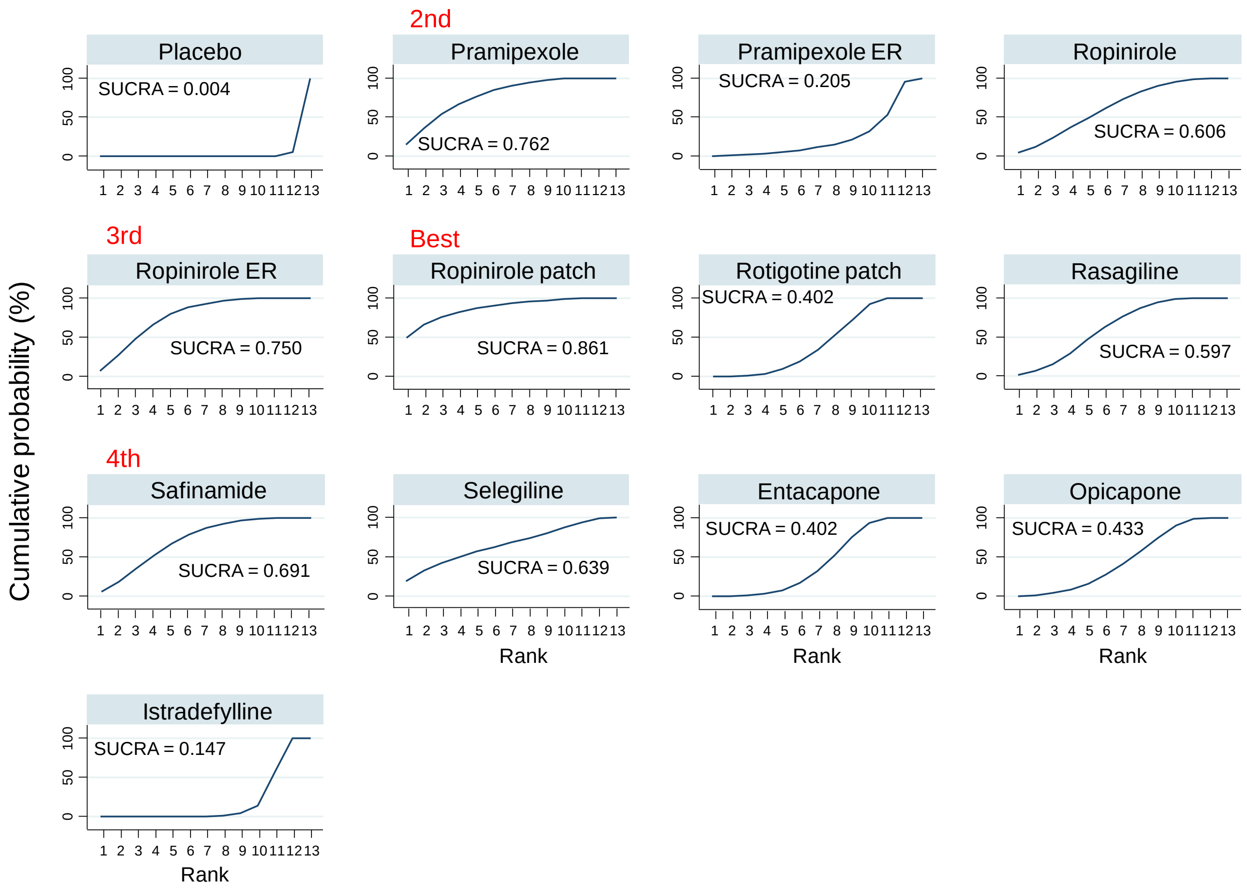

5B) Discontinuation due to all causes

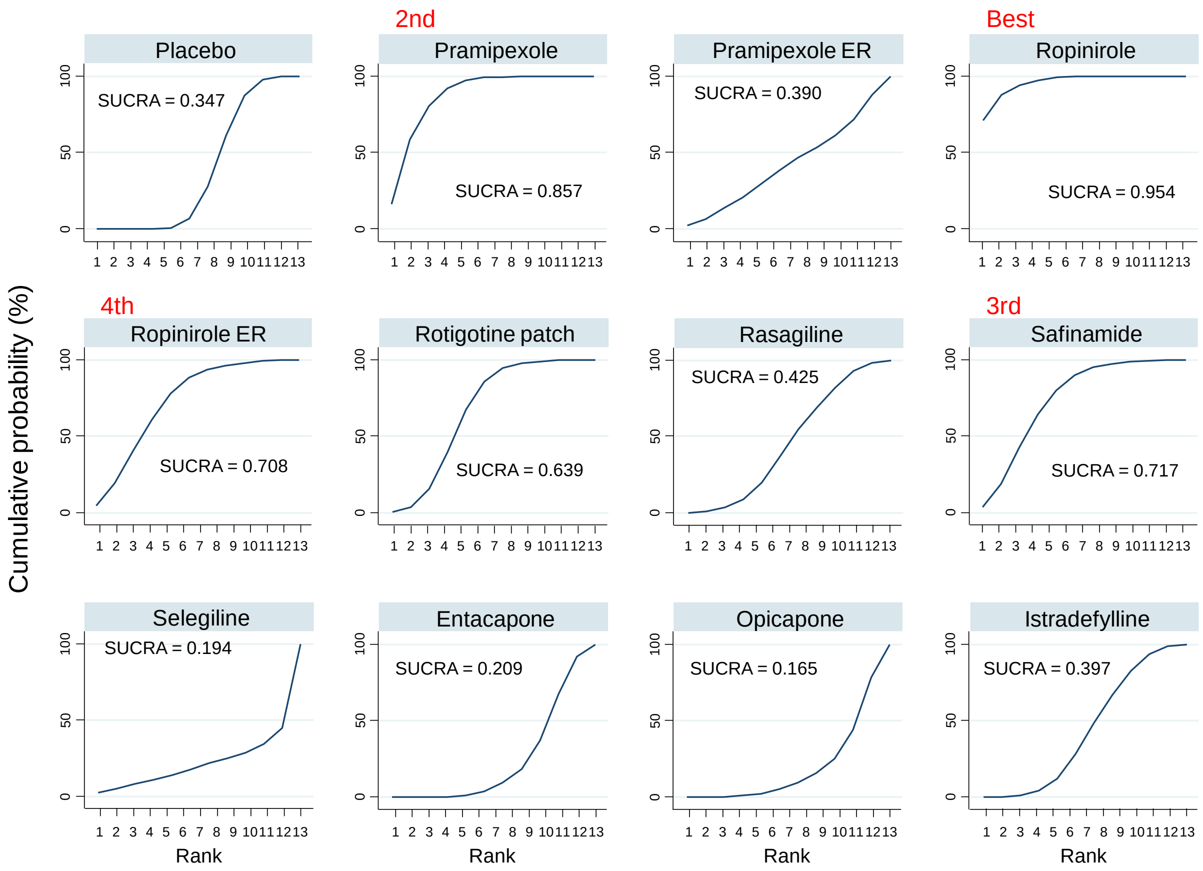

5C) Discontinuation due to AEs

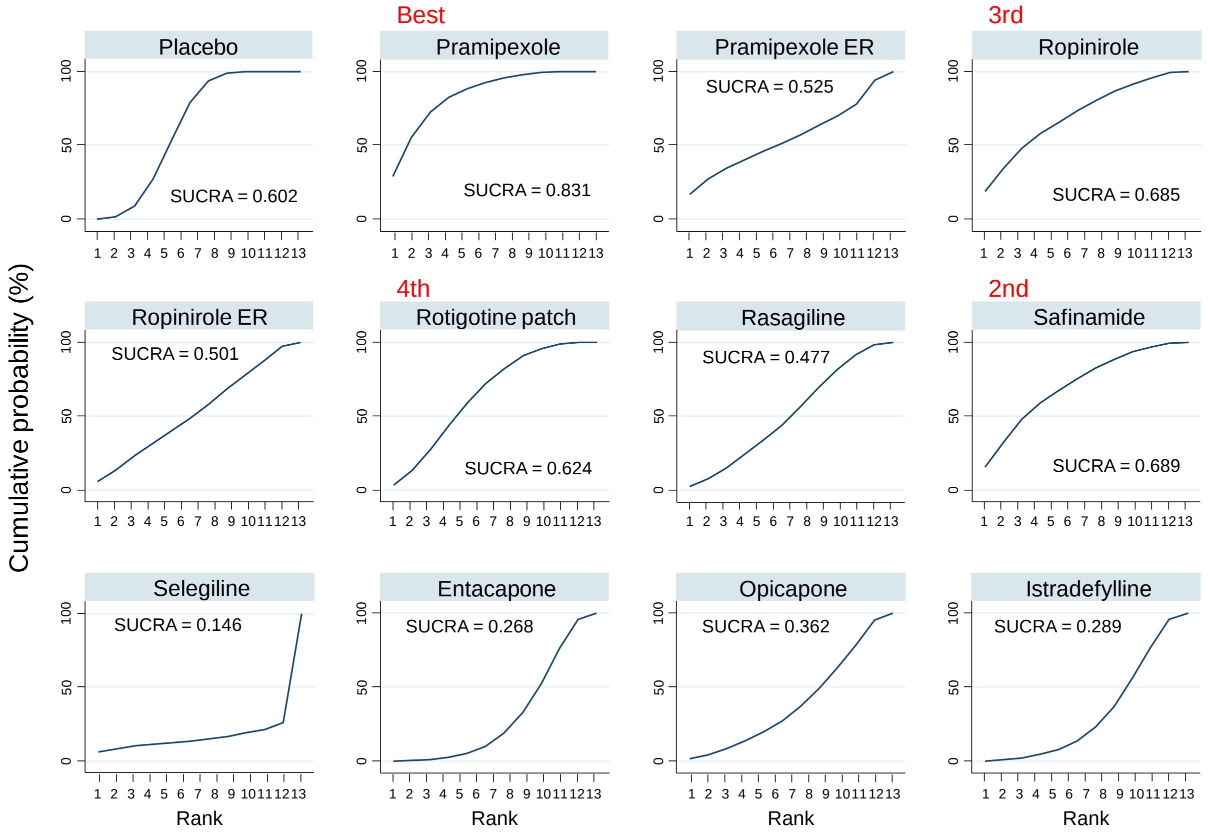

5D) AEs

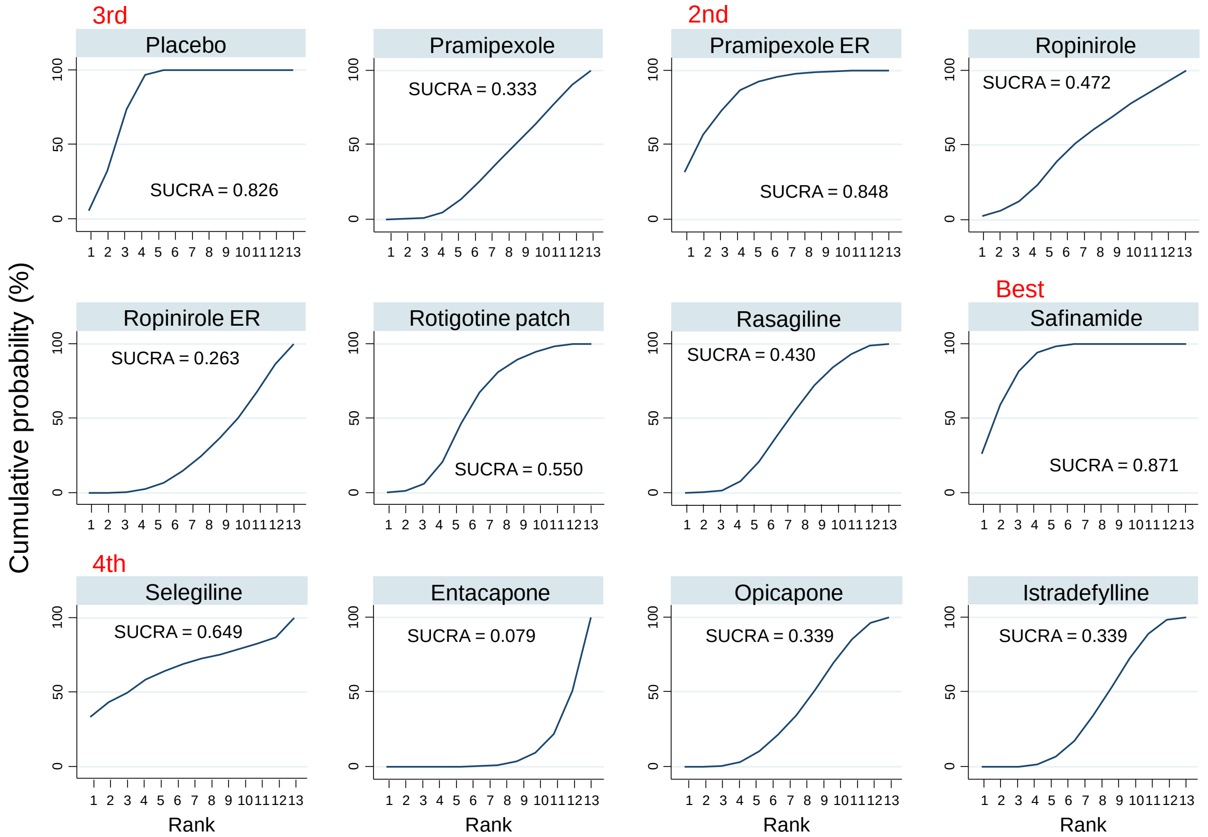

5E) Dyskinesia

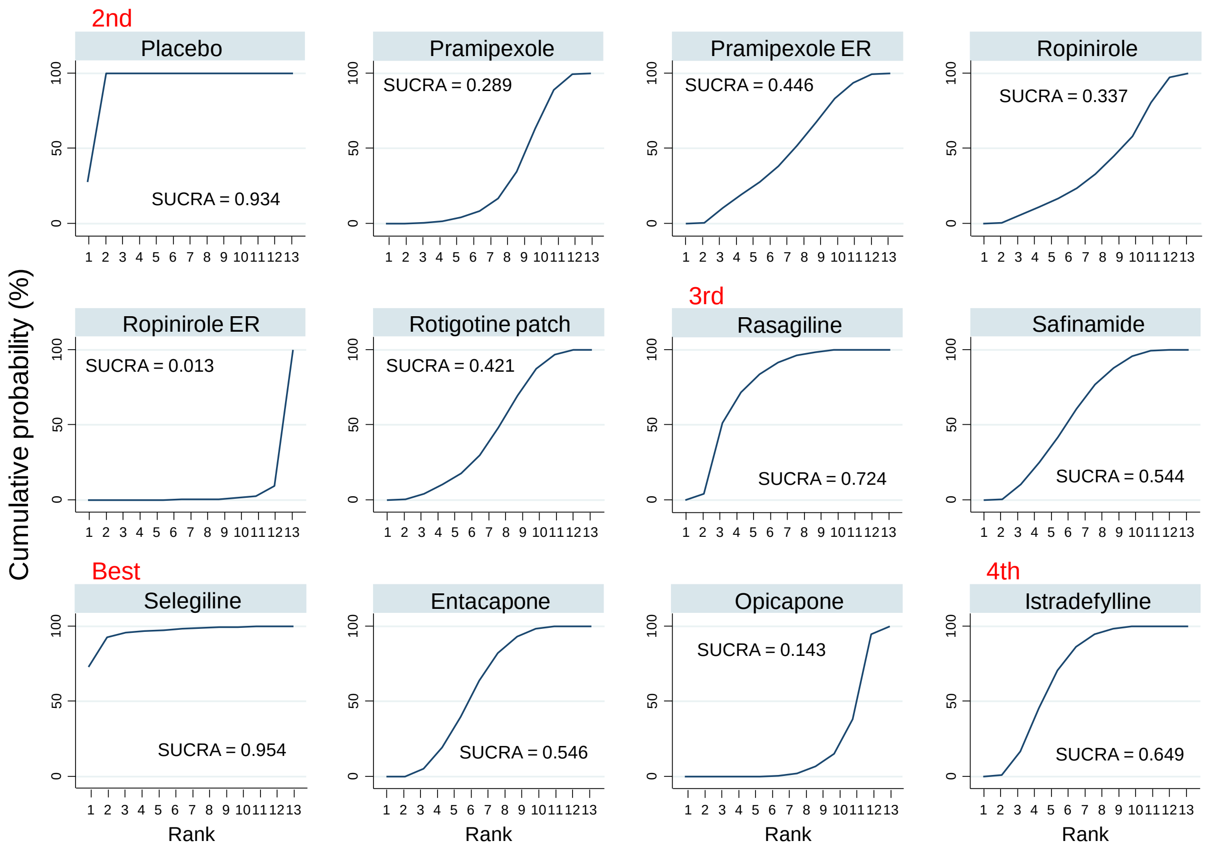

5F) Hallucination

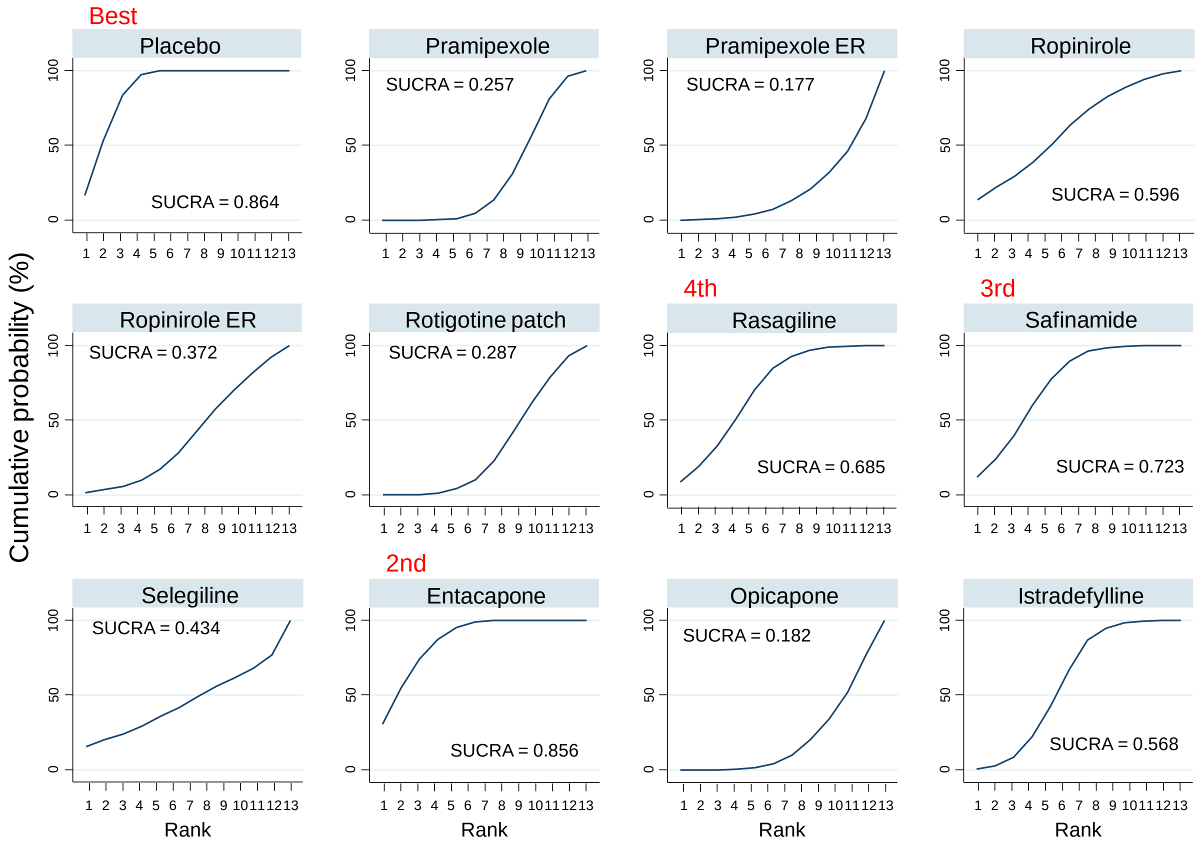

5G) Orthostatic hypotension

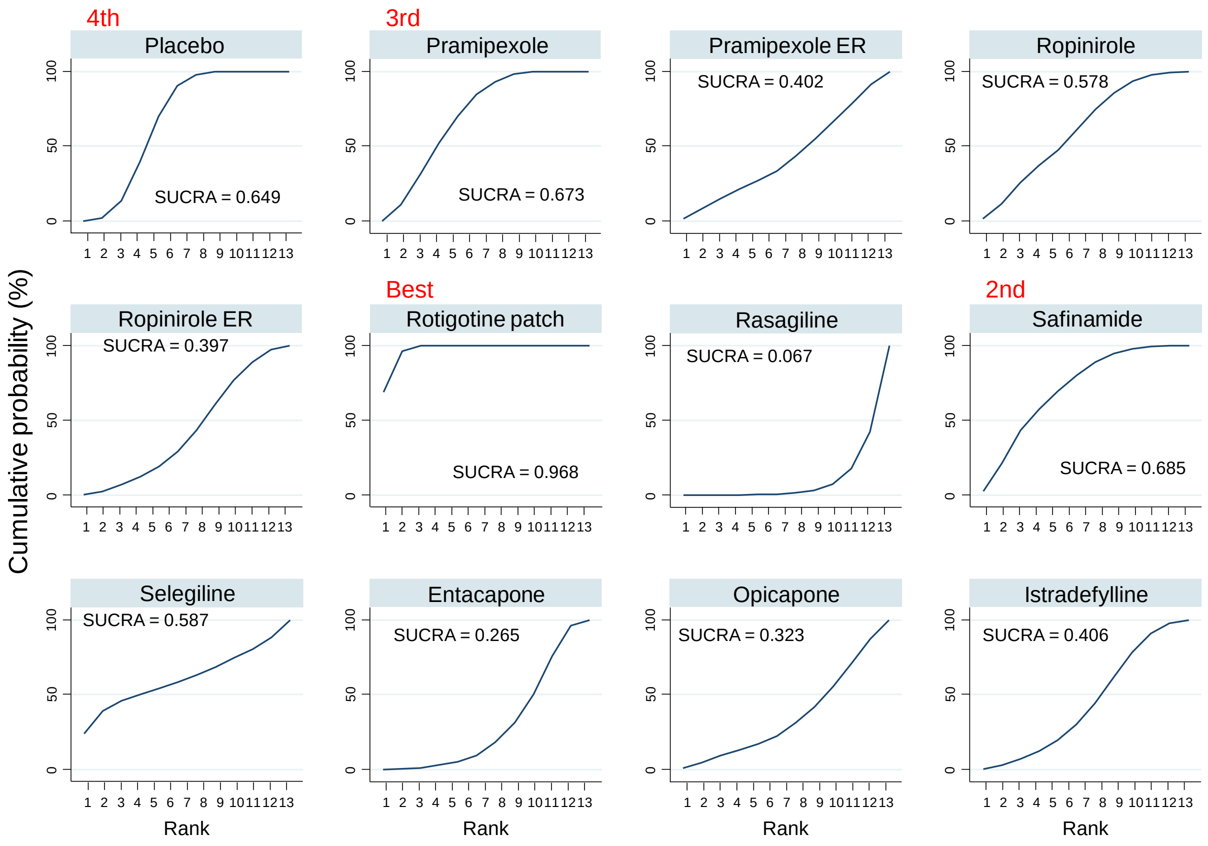

Supplementary Figure 5. Surface under the cumulative ranking curve (SUCRA) results

Cumulative ranking plots to show comparative efficacy, tolerability, and safety of treatments from network meta-analyses for each outcome. The greater SUCRA value from 0% to 100% indicates the better rank in efficacy, tolerability and safety evaluation.

Abbreviations: ER, extended release; ropinirole patch, ropinirole transdermal patch; rotigotine patch, rotigotine transdermal patch; AE, adverse event.

Supplementary Table 4. Search strategies

Sources	Search strategies
PubMed	("Parkinson disease"[MeSH Terms] OR ("Parkinson"[All Fields] AND "Disease"[All Fields]) OR "Parkinson disease"[All Fields] OR ("Parkinson's"[All Fields] AND "Disease"[All Fields]) OR "PD"[All Fields]) AND ("safinamide"[All Fields] OR "rasagiline"[All Fields] OR "selegiline"[All Fields] OR "pramipexole"[All Fields] OR "ropinirole"[All Fields] OR "rotigotine"[All Fields] OR "entacapone"[All Fields] OR "opicapone"[All Fields] OR "istradefylline"[All Fields]) AND ("randomized"[All Fields] OR "random"[All Fields] OR "randomly"[All Fields])
Embase	('Parkinson Disease'/exp OR ('Parkinson' AND 'Disease') OR 'Parkinson disease' OR ('Parkinson*' AND 'Disease') OR 'PD') AND ('safinamide' OR 'rasagiline' OR 'selegiline' OR 'pramipexole' OR 'ropinirole' OR 'rotigotine' OR 'entacapone' OR 'opicapone' OR 'istradefylline') AND ('randomly' OR 'randomized' OR 'random')
Cochrane Library	("MeSH descriptor: [Parkinson Disease] explode all trees" OR ("Parkinson" AND "Disease") OR "Parkinson disease" OR ("Parkinson's" AND "Disease") OR "PD") AND ("safinamide" OR "rasagiline" OR "selegiline" OR "pramipexole" OR "ropinirole" OR "rotigotine" OR "entacapone" OR "opicapone" OR "istradefylline") AND ("randomly" OR "randomized" OR "random")
